# Interferon‐induced transmembrane (IFITM) proteins at the tumour–immune interface: A four‐axis framework for their context‐dependent roles

**DOI:** 10.1002/ctm2.70767

**Published:** 2026-08-19

**Authors:** Zhe Liu, Jie Meng, Zengyan Lai, Fuqiu Li, Sha Lv

**Affiliations:** ^1^ Department of Dermatology The Second Hospital of Jilin University Changchun China; ^2^ Department of Ultrasonic Medicine The Second Hospital of Jilin University Changchun China

**Keywords:** cancer immunotherapy, IFITM proteins, immune resistance, interferon signalling, tumour immunity

## Abstract

**Background:**

The interferon‐induced transmembrane (IFITM) protein family, including IFITM1, IFITM2, IFITM3, and the less‐characterized IFITM5 and IFITM10, has emerged as a recurrent yet mechanistically complex group of players in cancer, reported as oncogenic in one setting and tumor‐restraining in another, immunosensitizing in one context and immunotherapy‐resistance‐promoting in the next. Rather than treating this literature as contradictory, we argue it is unresolved, and we organize it around a four‐axis framework defined by the interaction of member identity, interferon (IFN)‐input quality, cellular compartment, and tumor milieu.

**Main body:**

We first establish the foundational biology that compels this framework: family members are not interchangeable, occupying distinct subcellular compartments and partner networks; their abundance is set jointly by upstream IFN signaling and tumor‐cell‐intrinsic regulators; and post‐translational state gates their function independently of expression. We then refine the prevailing view of IFN signaling as monolithic, reformulating the direction of IFITM functional output around an acute‐versus‐chronic IFN distinction in which transient signaling drives major histocompatibility complex class I (MHC‐I) induction, antigen presentation, and immunogenicity, whereas sustained signaling promotes programmed death‐ligand 1 (PD‐L1)‐coupled adaptive immune resistance and engagement of the interferon‐stimulated gene resistance signature (ISG.RS). Surveying tumor‐cell‐intrinsic, immune‐cell‐intrinsic, stromal, and microbiota‐conditioned compartments across diverse malignancies, we show that the sign of any IFITM effect is set by where, when, and which member is expressed. The field's most prominent conflicts are reframed as falsifiable hypotheses with defined discriminating experiments.

**Conclusion:**

We conclude that translational maturity remains limited to biomarker discovery, and that compartment‐resolved, IFN‐quality‐stratified, immune‐competent studies, not further expression surveys, are the necessary next step.

**Key points:**

- Member, IFN quality, compartment, and milieu define IFITM cancer effects.
- IFITM paralogs engage distinct signaling networks across tumor and immune cells.
- Acute IFN favors MHC‐I immunogenicity; chronic IFN drives PD‐L1 resistance.
- Context resolves opposing IFITM effects into experimentally testable predictions.
- Near‐term translation favors biomarker stratification over direct IFITM targeting.

## Graphical abstract

1. Interferon‐induced transmembrane (IFITM) function in cancer is set by four contextual coordinates, not by family membership.
2. Acute interferon input drives major histocompatibility complex class I (MHC‐I)‐linked immunogenicity; chronic input drives programmed death‐ligand 1 (PD‐L1)‐coupled resistance.
3. An A–D evidence tier is applied to every claim, so correlative and causal studies are never equated.
4. The field's three central conflicts are restated as falsifiable hypotheses with defined experiments.

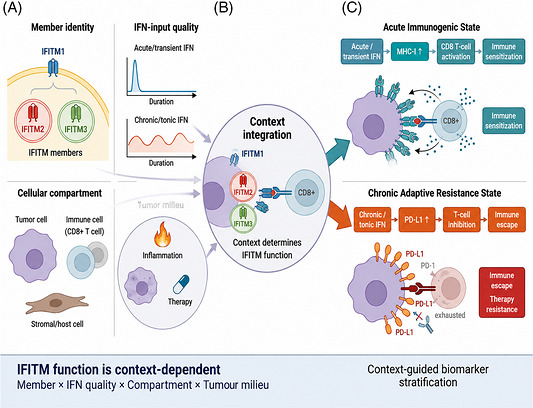

## 1 INTRODUCTION

Cancer remains one of the largest health burdens in the world.^1^, ^2^, ^3^ Although exceptional progress has been made in the fields of science and technology, there is a continued demand to enhance understanding of tumour biology and therapy.^4^ Resistance to chemotherapy and targeted agents further complicates the management, reinforcing the dire requirement of new therapeutic targets and approaches. With cancer continually evading standard treatments, discovery of novel molecular targets is vital to further enhancing patient outcomes and the field of cancer care. Interferon‐induced transmembrane (IFITM) proteins are a family of interferon (IFN)‐induced membrane proteins, which includes IFITM1, IFITM2, IFITM3, IFITM5 and IFITM10.^5^, ^6^, ^7^ Of these, IFITM1, IFITM2 and IFITM3 are IFN‐upregulated and are established effectors of the innate antiviral response, restricting entry of a broad range of viruses, whereas IFITM5 and IFITM10 are not IFN‐inducible, and no antiviral activity has been established for either member in mammalian systems, although characterisation of an IFITM10 orthologue in a non‐mammalian species has been reported (Section 2.1).^8^, ^9^ Immunity‐related IFITMs (IFITM1, 2 and 3) share a high degree of homology, but have different subcellular localisation and functions as revealed in recent studies.^10^, ^11^ Immunity‐associated IFITMs are potently induced by type I and type II IFNs and act predominantly as restriction factors that inhibit viral infections. They have an important function in the development of cancer, as well.^7^, ^12^, ^13^, ^14^

Increasing data indicate that IFITMs are involved in cancer progression. Expression of IFITM1, IFITM2 or IFITM3 is an unfavourable prognostic factor in several malignancies.^15^, ^16^, ^17^, ^18^, ^19^, ^20^ Therefore, IFITMs have been considered as a prognostic biomarker for specific cancer types. For example, abnormal IFITM1 protein levels were observed in gastroesophageal adenocarcinoma^21^ and upregulation of IFITM3 was associated with poor outcome in acute myeloid leukaemia (AML).^22^ The different IFITM members have a pivotal, context‐dependent (dual) role in tumour progression by influencing multiple hallmarks of tumour biology, including cell proliferation,^23^ cell migration,^24^ metastasis,^18^ epithelial–mesenchymal transition (EMT),^25^ tumour microenvironment (TME) remodelling,^26^ immune evasion,^27^ and chemoresistance.^23^ IFITM proteins contribute to cancer cell growth and survival through their interaction with key signalling pathways. For example, IFITM1 promotes cell survival and G1/S phase progression via the mitogen‐activated protein kinase (MAPK) pathway in pancreatic cancer.^23^ On the other hand, IFITM3 promotes gastric cancer stemness and chemoresistance through the Akt/forkhead box O3 (FOXO3)/c‐Myc axis.^28^

The IFITM cancer literature is therefore best characterised as unresolved rather than contradictory, a mechanistically detailed but conceptually fragmented literature in which the same family member is reported as oncogenic in one tumour and tumour‐restraining in another, immunosensitising in one cancer and immunotherapy‐resistance‐promoting in another, host‐protective in one compartment and tumour‐cell‐promoting in another. Prior focused reviews have surveyed this material on a per‐tumour or per‐family basis but have not articulated a framework that explains these apparent contradictions. This review aims to fill that gap. We introduce a four‐axis framework defined by the interaction of member identity, IFN‐input quality, cellular compartment and tumour milieu that organises the IFITM cancer literature without forcing it into family‐level statements (Section 2), refine the prevailing treatment of IFN signalling as monolithic by reformulating the direction of IFITM effects around the acute‐versus‐chronic IFN distinction (Section 6), and reframe the field's most prominent conflicts as testable hypotheses with defined discriminating experiments (Section 9). The aim is synthesis rather than cataloguing: to provide a framework that accommodates new findings and can be refined or refuted where it fails (Section 9).

## 2 IFITM BIOLOGY AND A FOUR‐AXIS FRAMEWORK FOR CONTEXT‐DEPENDENCE

This section summarises the molecular biology required to interpret Sections 2.1, 2.4, 3.2, 4.2, 5.1, 5.2 and defines the framework used to organise them. Three properties motivate that framework: (i) the immunity‐related members IFITM1, IFITM2 and IFITM3 are not functionally interchangeable; (ii) their expression is set jointly by upstream IFN input and by tumour‐cell‐intrinsic regulators; and (iii) their function is gated post‐translationally, independently of expression level. Together these properties define four axes, member identity, IFN‐input quality, cellular compartment and tumour milieu, that structure the remainder of this review.

*Literature search*. This is a structured narrative synthesis, not a systematic review. We searched PubMed and Web of Science (inception to April 2026), with Google Scholar as a supplement. Each IFITM member (IFITM1, IFITM2, IFITM3, IFITM5, IFITM10) was used as the primary search term and combined with (i) individual cancer types, (ii) immune‐cell compartments and (iii) the signalling nodes discussed here (Janus kinase/signal transducer and activator of transcription [JAK/STAT], nuclear factor kappa B [NF‐κB], major histocompatibility complex class I [MHC‐I]/human leukocyte antigen class I [HLA‐I], programmed death‐ligand 1 [PD‐L1]). Reference lists of retrieved primary articles and prior reviews were hand‐screened for additional studies. Studies in which an IFITM was reported only as one component of a broader macrophage IFN‐stimulated gene (ISG) signature, without member‐specific perturbation or functional analysis, were treated as contextual evidence and were not retained as stand‐alone mechanistic entries in the evidence tables.

*Public‐data statement*. No public dataset was newly downloaded, pooled or reanalysed for this review. Dataset accession numbers, cohort sizes and database‐derived findings reported in Tables 1, 2, 3, 4, 5 (Table 5 also reports TCGA, GEO, GSE165512 and cBioPortal‐derived findings), were extracted from the cited primary studies. Study‐specific preprocessing procedures, quality‐control criteria and access links should therefore be consulted in the corresponding source articles.

**TABLE 1:** Study‐level evidence for interferon‐induced transmembrane 1 (IFITM1) across solid tumours, grouped by organ system.

Cancer (subtype) and evidence base	IFITM member	Expression vs. normal/control	Functional role	Key mechanism/molecular axis	Axis context (IFN‐input · compartment)	Clinical, prognostic and therapy‐resistance findings	Tier	Refs.
**Breast carcinoma (triple‐negative breast cancer [TNBC]/inflammatory/ER‐positive subtypes)**								
Invasive breast cancer *BT20, MDA‐MB‐231; tissues + cell lines + xenograft*	IFITM1	↑ (with DDR2) in invasive tumours/lines	Oncogenic	Co‐expression with DDR2 drives proliferation, migration, invasion; suppresses apoptosis	IFN‐input: not IFN‐defined (DDR2 co‐receptor/adhesion node) Compartment: tumour cell	High DDR2/IFITM1 → poorer outcome and survival; dual knockdown exceeds single	**B**	^24^
ER‐positive breast cancer *MCF‐7 (wt p53), T47D (mut p53), TamR cells*	IFITM1	Expressed; paradoxically ↓ in tamoxifen‐resistant (TamR) vs. MCF‐7	Context‐dependent	JAK/STAT signalling; induction is p53‐dependent (IRF9/SOCS3)	IFN‐input: type I IFN‐linked (JAK/STAT–IRF9/SOCS3); IFN not manipulated, quality undefined Compartment: tumour cell	IFITM1 inhibition enhances tamoxifen sensitivity only in wt‐p53 cells → effect gated by p53 status	**C**	^67^
Inflammatory breast cancer (TNBC) *SUM149 vs. SUM190/MDA‐IBC‐3; functional assays*	IFITM1	Constitutively ↑ in SUM149 only (absent in SUM190, MDA‐IBC‐3)	Oncogenic	STAT2/BRG1‐dependent transcription; downstream of IFN‐α signalling	IFN‐input: type I IFN (IFN‐α–STAT2‐dependent transcription); quality undefined Compartment: tumour cell	Loss reduces migration, invasion, colony formation; candidate subtype‐specific target	**C**	^68^
Triple‐negative breast cancer *TMA/IHC + TCGA + lines + orthotopic and MIND in vivo + RNA‐seq*	IFITM1	↑ in TNBC samples (confirmed by TCGA)	Oncogenic	IFN‐α–NF‐κB crosstalk; NF‐κB (p65) is downstream effector; parthenolide suppresses IFITM1	IFN‐input: type I IFN (IFN‐α–NF‐κB crosstalk), sustained/tumour‐intrinsic Compartment: tumour cell	Knockout reduces tumour growth and invasion in vivo; proposed druggable axis	**B**	^48^
ER‐positive breast cancer (letrozole‐resistant) *tissues + cells + xenograft*	IFITM1	↑ in letrozole‐resistant tissues/cells (PITX2‐driven)	Oncogenic; resistance driver	PITX2 transactivates IFITM1 → potentiates IFN‐α‐induced AKT activation	IFN‐input: type I IFN (PITX2‐driven potentiation of IFN‐α‐induced AKT) Compartment: tumour cell	IFITM1 ablation restores letrozole sensitivity (endocrine resistance)	**B**	^79^
ER‐positive breast cancer (aromatase‐inhibitor‐resistant) *TMA (94 tumours) + MCF‐7:5C + orthotopic/MIND in vivo*	IFITM1	↑ in AI‐resistant MCF‐7:5C and resistant tumours	Oncogenic; resistance driver	Loss triggers JAK/STAT‐mediated p21 induction and nuclear localisation	IFN‐input: type I IFN‐associated (constitutive ISG state of AI‐resistant cells; IFN not manipulated) Compartment: tumour cell	Correlates with clinical stage and poor endocrine response; knockdown reduces growth/invasion in vivo	**B**	^49^
ER‐positive breast cancer (aromatase‐inhibitor‐resistant) *MCF‐7:5C vs. MCF‐7/T47D + IHC TMA*	IFITM1 (with PLSCR1)	Constitutively ↑ (co‐overexpressed with PLSCR1)	Oncogenic; resistance driver	Canonical IFN‐α–IFNAR–STAT1/2 axis drives ISG (incl. IFITM1) overexpression	IFN‐input: constitutive/chronic type I IFN (IFN‐α–IFNAR–STAT1/2 axis) Compartment: tumour cell	Suppression sensitises cells to estradiol‐induced death; ↑ p21/Bax/Noxa (AI resistance)	**C**	^80^
**Colorectal carcinoma**								
Colorectal cancer *metastatic lines + patient‐derived tumours*	IFITM1	↑ in metastatic CRC lines and tumours	Oncogenic	Dysregulates epithelial–mesenchymal transition (EMT) signature; effect partially reverted by Caveolin‐1 (CAV1)	IFN‐input: not IFN‐defined (EMT/CAV‐1 node) Compartment: tumour cell	High IFITM1 → poor prognosis; depletion reduces cell mobility — proposed prognostic marker/target	**C**	^16^
Colorectal cancer *cell lines + 229‐patient cohort*	IFITM1	↑; correlates with nodal/distant metastasis and advanced stage	Oncogenic	CAV1 identified as downstream mediator of IFITM1‐induced invasion (inverse IFITM1–CAV1 correlation)	IFN‐input: not IFN‐defined (CAV‐1‐dependent invasion) Compartment: tumour cell	Independent prognostic factor for poor survival	**C**	^17^
Colorectal cancer *mouse and patient‐derived organoids*	IFITM1	Induced by APC mutation; defines high vs. low subpopulations	Context‐dependent	APC/Wnt‐linked; IFITM1‐high cells are more proliferative and show reduced fibroblast‐EV uptake	IFN‐input: not IFN‐defined (APC/Wnt‐linked) Compartment: tumour cell; fibroblast extracellular‐vesicle crosstalk	IFITM1 inactivation enhances EV uptake, relevant to EV‐based therapeutic targeting and intratumour heterogeneity	**B**	^66^
**Lung carcinoma (non–small‐cell lung cancer [NSCLC] and small‐cell lung cancer [SCLC])**								
Non–small‐cell lung cancer *cell lines + 226 patient samples + in vivo*	IFITM1	↑; required for progression	Oncogenic	Maintains EGFR/SOX2 signalling axis; SOX2 re‐expression rescues IFITM1 loss	IFN‐input: not IFN‐defined (EGFR/SOX2 axis) Compartment: tumour cell	High IFITM1 → poor overall survival in adenocarcinoma but not squamous‐cell carcinoma (histology‐specific)	**B**	^69^
Small‐cell lung cancer *orthotopic xenograft + lines + patient tumours*	IFITM1	↑ in metastatic vs. orthotopic tumours	Oncogenic	Promotes distant metastatic colonisation (bone, adrenal); silencing suppresses metastasis	IFN‐input: not IFN‐defined (metastatic colonisation) Compartment: tumour cell	Drives experimental and orthotopic metastasis	**B**	^70^
Small‐cell lung cancer (cisplatin‐resistant) *TCGA + clinical samples + cells + in vivo*	IFITM1	↑ in tumour and in cisplatin‐resistant patients/cells	Oncogenic; resistance driver	Activates Wnt/β‐catenin pathway	IFN‐input: not IFN‐defined (Wnt/β‐catenin; chemotherapy‐selected state) Compartment: tumour cell	Silencing restores cisplatin chemosensitivity in vitro and in vivo	**B**	^78^
**Head and neck and oral‐cavity squamous carcinomas**								
Head and neck squamous cell carcinoma (SCC) *lines + cases (RT‐PCR/IHC) + invasion assays + microarray*	IFITM1	↑ in tumour (enriched at the invasive front of early‐invasive disease)	Oncogenic	Upregulates matrix metalloproteinases; promotes invasion	IFN‐input: not IFN‐defined (matrix‐metalloproteinase/invasion node) Compartment: tumour cell (invasive front)	Drives invasion at early stage; proposed therapeutic target	**C**	^71^
Laryngeal SCC *62 LSCC tissues + cells*	IFITM1	↑ in LSCC tissues and cells	Oncogenic	Osteopontin/NF‐κB‐dependent control of proliferation, migration, invasion	IFN‐input: not IFN‐defined (osteopontin/NF‐κB) Compartment: tumour cell	Proposed therapeutic target in LSCC	**C**	^72^
Oral cancer (radioresistant) *GEO/TCGA + 19 paired cases + cells + in vivo*	IFITM1	↑ (radioresistance‐associated; radiation induces IFITM1)	Oncogenic; resistance driver	Inhibition shifts STAT1/2/p21 up and STAT3/p‐p21 down; ↑ DNA damage and apoptosis	IFN‐input: radiation‐induced type I IFN/ISG program (IFITM1 induced by irradiation) Compartment: tumour cell	IFITM1 knockdown + radiotherapy cooperatively inhibit tumour growth (radiotherapy resistance)	**B**	^81^
Oral SCC (radioresistant, TME‐driven) *CAFs/exosomes + cells + in vivo*	IFITM1 (downstream of CAF‐exosomal lncRNA RORA‐AS1)	↑ under CAF‐exosome/RORA‐AS1 exposure + irradiation	Oncogenic; resistance driver	RORA‐AS1 binds IFITM1 RNA → IFITM1/STAT axis; knockdown ↑ p‐STAT1/p21/p53, ↓ p‐STAT3/IFITM1	IFN‐input: stromal‐conditioned (CAF‐exosomal lncRNA RORA‐AS1 → IFITM1/STAT axis); IFN not directly manipulated Compartment: cancer‐associated fibroblast (stromal) → tumour cell	Microenvironment‐mediated radiotherapy resistance; RORA‐AS1 as candidate biomarker	**B**	^82^
**Gastrointestinal carcinomas (gastric and pancreatic)**								
Gastric cancer *lines + 35 + 151 patient tissues (qRT‐PCR/WB/IHC)*	IFITM1	↑; associated with Lauren intestinal type and differentiated adenocarcinoma	Oncogenic	Epigenetic control — promoter hypermethylation silences IFITM1 in low‐expressers	IFN‐input: not IFN‐defined (promoter‐methylation control) Compartment: tumour cell	Silencing reduces migration/invasion; expression linked to clinicopathology and prognosis	**C**	^74^
Pancreatic cancer *PANC‐1, ASPC‐1 (shRNA)*	IFITM1	Functionally required (knockdown model)	Oncogenic	Sustains MAPK signalling (p‐ERK, p‐P38, p‐JNK); knockdown causes G1/S arrest and apoptosis	IFN‐input: not IFN‐defined (MAPK node) Compartment: tumour cell	Proposed therapeutic target	**C**	^23^
Pancreatic cancer *5 expression profiles + 90‐patient IHC + cells*	IFITM1	↑ in tumour vs. normal duct epithelium	Oncogenic	Network‐module hub; silencing reduces tumourigenicity; correlates with immune‐cell infiltration	IFN‐input: not IFN‐defined (computational hub) Compartment: tumour cell, with bulk immune‐infiltrate correlation	High IFITM1 → poor survival; independent prognostic factor (multivariate Cox)	**C**	^85^
**Gynaecological carcinomas (ovarian and cervical)**								
Ovarian cancer *intraperitoneal xenograft + SK‐OV‐3*	IFITM1	↑ in metastatic implants	Oncogenic	Promoter hypomethylation (epigenetic) elevates IFITM1; ↑ migration/invasion	IFN‐input: not IFN‐defined (promoter hypomethylation) Compartment: tumour cell	IFITM1 promoter methylation status proposed as a metastasis biomarker	**B**	^73^
Ovarian clear‐cell carcinoma *DIA‐MS proteomics (3 cohorts) + PRM + IHC*	IFITM1	Identified as a prognostic protein (direction not functionally tested)	Prognostic biomarker	Proteomic correlate of recurrence‐free survival (no functional manipulation)	IFN‐input: not IFN‐defined (proteomic correlate; no perturbation) Compartment: tumour cell	Robust prognostic marker for recurrence/survival in CCOC (correlative)	**C**	^86^
Cervical cancer *surface‐proteomics (MS) + RT‐qPCR + functional assays*	IFITM1 and IFITM3 (both tested)	IFITMs frequently overexpressed in cervical carcinoma cells	Oncogenic; immune‐modulatory	Reshapes the surfaceome (endocytosis, lysosomal transport, adhesion, antigen presentation, immune response); IFN‐γ‐responsive	IFN‐input: IFN‐γ (type II) responsiveness assessed; acute vs. chronic not defined Compartment: tumour cell, with tumour–immune‐cell interaction readouts	IFITM1 and IFITM3 affect adhesion, migration, invasion and tumour–immune‐cell interaction. Cf.,^75^, opposite direction in the same lineage; see Section 9	**C**	^77^
Cervical squamous cell carcinoma *tumour vs. adjacent/normal cervical epithelium + cells*	IFITM1	↓ (downregulated) vs. adjacent/normal cervical epithelium	Tumour‐suppressive/growth‐inhibitory	IFITM1 restoration restrains proliferation, consistent with a retained antiviral/growth‐inhibitory program	IFN‐input: not IFN‐defined (IFN not manipulated) Compartment: tumour cell	Directionally opposite to^77^ in the same lineage; comparator is virally exposed, IFN‐conditioned mucosa, so the apparent loss may partly reflect an elevated control baseline (Section 9)	**C**	^75^
**Genitourinary carcinomas (prostate and bladder)**								
Prostate adenocarcinoma *in silico only (GSE32571/public databases)*	IFITM1	↓ (downregulated) in PRAD	Tumour‐suppressive/favourable	IFITM1‐associated genes enriched in Th1/Th2/Th17 differentiation, NK cytotoxicity, DC activation; correlates with 22 immune‐cell types	IFN‐input: not IFN‐defined (in silico immune correlation) Compartment: tumour plus immune infiltrate (computational deconvolution)	LOW IFITM1 → poor prognosis (opposite direction). Caveat: bioinformatics‐only, no functional validation	**C**	^135^
Bladder cancer *bladder lines + IFN‐α/adenoviral (IFNα‐2b) delivery*	type I IFN/ISG program (IFITM1 as one candidate marker)	Constitutive type I IFN‐pathway activation	Resistance biomarker	Constitutive type I IFN signalling marks resistance to IFN‐α‐induced cytotoxicity	IFN‐input: constitutive/chronic type I IFN activation (resistance to exogenous IFN‐α cytotoxicity) Compartment: tumour cell	Candidate resistance biomarkers for IFN‐based therapy (e.g., nadofaragene firadenovec). Caveat: IFITM1‐specific evidence is indirect	**C**	^84^
**Anal squamous‐cell carcinoma**								
Anal SCC (chemoradiotherapy) *130 IHC + 98 RNA‐seq + blood immune profiling*	IFITM1	↑ (mRNA and protein) in poor responders to CRT	Resistance‐associated (prognostic)	Inflammatory/interferon program enriched (IFNγ, IFN‐α, TNF‐α–NF‐κB, EMT) in poor responders	IFN‐input: chronic inflammatory (IFN‐α/IFN‐γ, TNF‐α–NF‐κB program in non‐responders) Compartment: tumour cell, with peripheral‐blood immune profiling	IFITM1 associated with reduced freedom from locoregional failure (FFLF) and distant metastasis (FFDM)	**C**	^83^
**Peripheral nerve‐sheath tumour (**neurofibromatosis type 1‐associated malignant peripheral nerve sheath tumour [NF1‐MPNST])								
NF1‐associated MPNST *patient MPNST vs. plexiform neurofibroma + cells + xenograft*	IFITM1	↓ (downregulated) in malignant MPNST vs. benign plexiform neurofibroma	Tumour‐suppressive	IFITM1 overexpression lowers active Ras (GTP‐Ras) and ERK1/2 phosphorylation; IFN‐γ induces IFITM1	IFN‐input: IFN‐γ (type II), exogenous exposure Compartment: tumour cell	IFN‐γ suppresses xenograft growth via IFITM1 (opposite direction). Functional + in vivo evidence	**B**	^76^

**Axis mapping**: The four contextual axes are resolved across the table: Member identity (column ‘IFITM member’), Tumour‐milieu/context (tissue lineage and subtype in column ‘Cancer (subtype) and evidence base’), and IFN‐input quality and Cellular compartment (column ‘Axis context’). *‘Not IFN‐defined*’ denotes studies in which IFITM1 was examined as a signalling/EMT node without a manipulated or specified IFN regime; acute versus chronic is assigned only where the primary study defined the IFN input.

**Evidence tiers**: A: Member‐specific causal evidence from in vivo functional perturbation (genetic deletion, knockdown or targeted pharmacological intervention), corroborated in immune‐competent models and/or independent human cohorts; B: In vivo or robust mechanistic evidence demonstrating a partial causal chain, including xenograft, CRISPR‐based or other mouse‐model studies, as well as single‐cohort evidence supported by functional experiments; C: In vitro, correlative, marker‐level or computational evidence lacking in vivo functional validation; D: Preliminary biomarker‐ or association‐level evidence from unvalidated single cohorts, conference abstracts or studies lacking both external validation and functional support. Conference abstracts are identified as such in the evidence‐base column and are assigned Tier D unless functional evidence is available. ‘—’ marks context‐only rows that do not provide IFITM‐specific functional or associative data (the study does not name, measure or manipulate an IFITM) and are included solely to frame the upstream IFN‐input or checkpoint context; these are deliberately not assigned an IFITM evidence tier so that different evidence types are not treated as equivalent support. Preprints are identified as such in the evidence‐base column and are treated as provisional evidence.

**Symbols**: ↑, increased/upregulated; ↓, decreased/downregulated; →, leads to.

**TABLE 2:** Study‐level evidence for interferon‐induced transmembrane 3 (IFITM3) across cancers.

Cancer (subtype) and evidence base	IFITM member	Expression/variant vs. normal	Functional role	Key mechanism/molecular axis	Axis context (IFN‐input · compartment)	Clinical, prognostic and therapy‐resistance findings	Tier	Refs.
**Hepatocellular carcinoma (genetic‐variant, non‐coding‐RNA and biomarker studies)**								
HCC (HCV‐related) *50 HCC vs. 50 HCV; DNA sequencing + ELISA*	IFITM3	rs12252‐CC genotype and protein ↑ in HCC vs. HCV controls	Genetic risk variant	IFITM3 rs12252 single‐nucleotide polymorphism (germline)	IFN‐input: chronic viral (HCV) disease context; not experimentally manipulated Compartment: tumour cell (germline‐variant carrier)	CC genotype associated with HCC risk (OR = 2.70, *p* = .041); proposed diagnostic marker	**D**	^87^
HCC (HCV‐related) *100 HCC vs. 100 HCV; PCR‐RFLP, sequencing, ELISA*	IFITM3 (with MMP‐9)	IFITM3 C allele and MMP‐9 T allele more frequent in patients	Genetic risk variant	Co‐occurring IFITM3 and MMP‐9 gene polymorphisms	IFN‐input: chronic viral (HCV) disease context; not experimentally manipulated Compartment: tumour cell (germline‐variant carrier)	IFITM3 CC genotype OR = 2.43; MMP‐9 TT OR = 2.63, linked to HCC occurrence/development	**D**	^88^
HCC *tissues + cell lines + dual‐luciferase*	IFITM3	↑ IFITM3 and ↓ miR‐29a in HCC (inversely correlated)	Oncogenic	IFITM3 is a direct functional target of miR‐29a	IFN‐input: not IFN‐defined (non‐coding‐RNA control) Compartment: tumour cell	Associated with tumour size, multifocality, venous invasion; high IFITM3/low miR‐29a → poor prognosis	**C**	^92^
HCC *cells + in vivo + rescue experiments*	IFITM3	↑; positively correlated with c‐MYC	Oncogenic	IFITM3–ERK1/2–c‐MYC regulatory circuit	IFN‐input: not IFN‐defined (ERK1/2–c‐MYC output) Compartment: tumour cell	Knockdown reduces c‐MYC and proliferation in vitro and in vivo	**B**	^95^
HCC *expression databases + tissues + PLC/PRF/5 (lentiviral) + RNA‐seq*	IFITM3	↑ vs. adjacent normal; higher in low‐differentiation/metastatic tumours	Oncogenic	PI3K/AKT/mTOR pathway; reduces vimentin (epithelial–mesenchymal transition [EMT]); higher RNA in rs12252‐CC genotype	IFN‐input: chronic viral disease context; not experimentally manipulated Compartment: tumour cell	Knockdown inhibits proliferation/migration; expression tracks differentiation and metastatic potential	**C**	^91^
HCC (HBV‐related) *156 HCC vs. 180 HBV/cirrhosis; PCR genotyping*	IFITM3	rs12252‐CC over‐represented in HCC, especially low differentiation	Genetic risk variant	IFITM3 rs12252 germline variant	IFN‐input: chronic viral (HBV) disease context; not experimentally manipulated Compartment: tumour cell (germline‐variant carrier)	CC genotype: larger tumours, more vascular thrombosis, lower differentiation, higher 5‐year relapse	**D**	^89^
HCC and cirrhosis (HCV‐related) *case‐control, 1350 subjects; TaqMan RT‐PCR*	IFITM3 (with IFNL4)	rs12252 CC ↑ in cirrhosis; CT+CC ↑ in HCC vs. healthy	Genetic risk variant	IFITM3 rs12252 and IFNL4 rs12979860 polymorphisms	IFN‐input: chronic viral (HCV) disease context; IFNL4 variant co‐assessed, IFN not manipulated Compartment: tumour cell/host germline	Both variants linked to development of cirrhosis and HCC in chronic HCV infection	**D**	^90^
HCC *tissues + cells; dual‐luciferase; CCK‐8/transwell*	IFITM3	IFITM3 elevated downstream of lncRNA CKMT2‐AS1/low miR‐142‐5p	Oncogenic	CKMT2‐AS1 sponges miR‐142‐5p → de‐represses IFITM3 (ceRNA network)	IFN‐input: not IFN‐defined (competing‐endogenous‐RNA network) Compartment: tumour cell	Axis promotes proliferation, migration and invasion of HCC cells	**C**	^93^
HCC *65 patients + cells + xenograft + FISH/luciferase*	IFITM3	TUG1 and IFITM3 ↑, miR‐29a ↓ in HCC	Oncogenic	TUG1 competitively binds miR‐29a → positively regulates IFITM3 (ceRNA)	IFN‐input: not IFN‐defined (competing‐endogenous‐RNA network) Compartment: tumour cell	TUG1 knockdown reduces invasion/migration/proliferation, promotes apoptosis; prognostic relevance	**B**	^94^
Advanced HCC (CNLC III; HAIC + sintilimab + bevacizumab biosimilar) *conference abstract (AACR 2025; not peer‐reviewed); 65 patients + scRNA/bulk RNA‐seq + machine learning*	IFITM3	High tumoural and exosomal IFITM3	Predictive/prognostic biomarker	Microvascular‐invasion hub gene; detectable in blood‐borne exosomes	IFN‐input: not IFN‐defined (checkpoint‐treated cohort; IFN quality not assessed) Compartment: tumour cell; blood‐borne exosomes	High IFITM3 → shorter survival (*p* < .001); candidate non‐invasive marker for therapy stratification. Caveat: conference‐abstract evidence, not peer‐reviewed; graded Tier D per the abstract rule (Section 2)	**D**	^98^
**Gastrointestinal carcinomas (gastric and colorectal)**								
Gastric cancer *quantitative proteomics + clinical cohort + functional assays*	IFITM3	↑ in tumour vs. adjacent epithelium; correlates with advanced stage	Oncogenic; chemoresistance and stemness	PIP3 scaffold; associates with MET/AKT → suppresses FOXO3 → induces c‐MYC (MET/AKT/FOXO3/c‐MYC axis)	IFN‐input: not IFN‐defined (MET/AKT scaffold) Compartment: tumour cell	Promotes growth, metastasis, cancer stemness and chemoresistance; depletion blunts HGF/MET‐driven growth	**B**	^28^
Gastric cancer *cell lines + tissues (RT‐qPCR/WB/IHC)*	IFITM3	↑; correlates with differentiation, nodal/distant metastasis, TNM stage	Oncogenic	Wnt/β‐catenin signalling; silencing reverses EMT and lowers MMP‐2/MMP‐9	IFN‐input: not IFN‐defined (Wnt/β‐catenin, EMT) Compartment: tumour cell	Knockdown suppresses migration/invasion/proliferation, G0/G1 arrest; proposed target	**C**	^96^
Colon cancer *TMA of 203 patients + cells + xenograft + transgenic mice*	IFITM3	↑ in tumours, highest in nodal metastases; inverse to KLF4	Oncogenic	Direct transcriptional target of tumour‐suppressor KLF4 (KLF4 represses IFITM3 promoter)	IFN‐input: not IFN‐defined (KLF4 transcriptional repression) Compartment: tumour cell; intestinal epithelium in Villin‐Cre Klf4‐null mice	Independent prognostic factor for disease‐free survival; knockdown suppresses growth/metastasis	**B**	^15^
Colon cancer *expression microarray + TMA/IHC validation*	IFITM3	↑ in colon cancer; significant increase already in adenoma vs. normal	Oncogenic	Candidate marker of early colon carcinogenesis	IFN‐input: not IFN‐defined (expression profiling) Compartment: tumour cell (adenoma and carcinoma epithelium)	Supports a role in early colon‐cancer development	**C**	^97^
**Head and neck (oral squamous‐cell carcinoma)**								
Oral squamous cell carcinoma (SCC) *tissues (qRT‐PCR/IHC) + siRNA knockdown + WB*	IFITM3	↑ in >79% of primary OSCCs	Oncogenic	Regulates CCND1–CDK4/6–pRB axis (lowers CCND1, CDK4, RB phosphorylation)	IFN‐input: not IFN‐defined (cell‐cycle axis) Compartment: tumour cell	Knockdown causes cell‐cycle arrest, senescence and apoptosis; candidate target	**C**	^100^
**Breast carcinoma**								
Invasive breast cancer *64 patients (IHC) + shRNA in cell lines*	IFITM3	↑ in invasive breast cancer vs. normal; associated with ER/PR status	Oncogenic	Drives G0/G1 → S transition (cell‐cycle control)	IFN‐input: not IFN‐defined (G0/G1–S control) Compartment: tumour cell	Knockdown reduces viability, growth and colony formation; candidate target	**C**	^101^
**Central nervous system (glioblastoma)**								
Glioblastoma multiforme *TCGA/CGGA + glioblastoma stem cells + endothelial co‐culture*	IFITM3	↑ in GBM; predictive of adverse outcome	Oncogenic; pro‐angiogenic	GSC‐derived IFITM3 → Jak2/STAT3 activation → bFGF secretion → angiogenesis	IFN‐input: not IFN‐defined (JAK/STAT3–bFGF) Compartment: glioblastoma stem cell → endothelial crosstalk (paracrine)	Enhances tumour angiogenesis; new angiogenic mechanism	**C**	^99^
**Genitourinary carcinomas (prostate and renal)**								
Prostate cancer (bone metastasis) *cells + knockdown/overexpression + microarray*	IFITM3	Functional driver of bone metastasis	Oncogenic; pro‐metastatic	Binds Smad4 → activates TGF‐β–Smads–MAPK; reverses EMT and lowers FGFs/PTHrP on knockdown	IFN‐input: not IFN‐defined (TGF‐β–SMAD4/MAPK) Compartment: tumour cell (bone‐metastatic niche)	Promotes proliferation, invasion and bone migration. Contrast: low IFITM1 is associated with poor prognosis in prostate adenocarcinoma in an in silico analysis (Tier C); see Table 1 and Section 8.1	**C**	^18^
Clear‐cell renal cell carcinoma (TKI‐resistant) *resistant cohort + public dataset + sunitinib‐resistant lines + xenograft*	IFITM3	↑ in post‐TKI samples and in ccRCC vs. normal kidney	Oncogenic; resistance driver	Activates TRAF6 and MAPK/AP‐1 pathways	IFN‐input: not IFN‐defined (TRAF6/MAPK/AP‐1); post‐TKI therapy‐selected state Compartment: tumour cell	Confers acquired sunitinib (VEGF‐TKI) resistance; IFITM3 inhibition restores sensitivity, candidate target	**B**	^47^
**Thoracic (EGFR‐mutant non–small‐cell lung cancer)**								
EGFR‐mutant NSCLC (osimertinib‐resistant) *clinical specimens + PC‐9/H1975 + spatial transcriptomics + xenograft*	IFITM3	↑ in poor responders; induced by TME‐derived cytokines during treatment	Oncogenic; resistance driver	IFITM3–MET interaction → AKT pathway activation	IFN‐input: TME cytokine‐induced during treatment (therapy‐associated, sustained); IFN quality not resolved Compartment: tumour cell, with TME cytokine input	IFITM3‐positive tumours show shorter PFS on osimertinib; MET inhibition suppresses resistance in vivo	**B**	^60^
**Haematological malignancies (acute myeloid and chronic myeloid leukaemia)**								
Acute myeloid leukaemia *TCGA, 155 patients; survival analysis*	IFITM family (IFITM3 focus)	High IFITM3 expression	Adverse prognostic biomarker	Prognostic association within IFITM1–5 screen	IFN‐input: not IFN‐defined (prognostic association) Compartment: leukaemic cell (bulk cohort)	High IFITM3 → shorter EFS/OS in chemotherapy‐only patients (independent risk factor); no effect after allo‐HSCT	**D**	^22^
Acute myeloid leukaemia *KG‐1a (LV‐shIFITM3) + NOD/SCID mice*	IFITM3	IFITM3 functionally required (knockdown model)	Oncogenic; therapeutic target	Knockdown → cell‐cycle arrest and apoptosis; iron‐oxide nanoparticles + cytarabine further lower IFITM3 and raise ROS	IFN‐input: not IFN‐defined (pharmacological downregulation) Compartment: leukaemic cell	IONP + Ara‐C combination suppresses AML in vitro and in vivo, IFITM3 as actionable target	**B**	^102^
Chronic myeloid leukaemia (imatinib‐resistant) *K562IR exosomes; label‐free proteomics + flow cytometry*	IFITM3 (with CD146, CD36)	↑ in imatinib‐resistant K562 exosomes and cells (surface‐localised)	Resistance‐associated biomarker	Exosomal transfer of resistance‐associated membrane proteins to sensitive cells	IFN‐input: not IFN‐defined (exosomal membrane‐protein transfer) Compartment: leukaemic cell → recipient sensitive leukaemic cell	Candidate exosomal marker of TKI resistance (CD146 most validated functionally)	**C**	^103^
Acute myeloid leukaemia *single‐cell + bulk multi‐omics (SCENIC); prognostic modelling*	IFITM1 (not IFITM3)	IFITM1 included in a seven‐gene endoplasmic‐reticulum‐stress signature	Prognostic‐signature component	Gene‐regulatory‐network/ER‐stress‐based signature (MPO, HOPX, CST3, ITM2A, CD96, IFITM1, SPINK2)	IFN‐input: not IFN‐defined (computational signature) Compartment: leukaemic cell (bulk and single‐cell, in silico)	Nomogram predicts OS (AUC .72–.80). Caveat: concerns IFITM1, not IFITM3, and is one of seven genes, indirect	**D**	^104^

**Axis mapping**: The four contextual axes are resolved across the table: Member identity (column ‘IFITM member’), Tumour‐milieu/context (tissue lineage and subtype in column ‘Cancer (subtype) and evidence base’), and IFN‐input quality and Cellular compartment (column ‘Axis context’). ‘Not IFN‐defined’ denotes studies in which IFITM3 was examined as a kinase‐, scaffold‐ or non‐coding‐RNA‐regulated node without a manipulated or specified IFN regime. ‘Chronic viral (HCV/HBV) disease context’ denotes germline‐variant studies conducted in chronically infected populations in which IFN input was not experimentally manipulated. Acute versus chronic is assigned only where the primary study defined the IFN input.

**Evidence tiers**: A: Member‐specific causal evidence from in vivo functional perturbation (genetic deletion, knockdown or targeted pharmacological intervention), corroborated in immune‐competent models and/or independent human cohorts; B: In vivo or robust mechanistic evidence demonstrating a partial causal chain, including xenograft, CRISPR‐based or other mouse‐model studies, as well as single‐cohort evidence supported by functional experiments; C: In vitro, correlative, marker‐level or computational evidence lacking in vivo functional validation; D: Preliminary biomarker‐ or association‐level evidence from unvalidated single cohorts, conference abstracts or studies lacking both external validation and functional support. Conference abstracts are identified as such in the evidence‐base column and are assigned Tier D unless functional evidence is available. ‘—’ marks context‐only rows that do not provide IFITM‐specific functional or associative data (the study does not name, measure or manipulate an IFITM) and are included solely to frame the upstream IFN‐input or checkpoint context; these are deliberately not assigned an IFITM evidence tier so that different evidence types are not treated as equivalent support. Preprints are identified as such in the evidence‐base column and are treated as provisional evidence.

**Symbols**: ↑, increased/upregulated; ↓, decreased/downregulated; →, leads to.

**TABLE 3:** Study‐level evidence for interferon‐induced transmembrane 2 (IFITM2), IFITM5 and IFITM10 across cancers.

Cancer (subtype) and evidence base	IFITM member	Expression/context vs. normal	Functional role	Key mechanism/molecular axis	Axis context (IFN‐input · compartment)	Clinical, prognostic and translational findings	Tier	Refs.
**IFITM2 across solid tumours**								
Clear‐cell renal cell carcinoma *tissues + cells + TCGA 538 cases + lymphatic‐endothelial co‐culture*	IFITM2	↑ in tumour vs. normal; high IFITM2 → shorter survival	Oncogenic; pro‐lymphangiogenic	Silencing impairs ccRCC migration/invasion; conditioned media from IFITM2‐silenced ccRCC suppresses human lymphatic endothelial cell proliferation, migration and tube formation	IFN‐input: not IFN‐defined (IFN‐inducible member, IFN not manipulated) Compartment: tumour cell; lymphatic‐endothelial crosstalk (paracrine)	Promising target and predictive marker for ccRCC malignancy	**C**	^107^
Colorectal cancer *TCGA + WB + siRNA in SW480 and HCT116*	IFITM2	↑ in CRC tissues and cells; linked to N, M and pathologic stage	Oncogenic	GSEA and WB confirm activation of the PI3K/AKT pathway	IFN‐input: not IFN‐defined (PI3K/AKT) Compartment: tumour cell	Significant impact on survival; knockdown reduces proliferation, migration, invasion	**C**	^105^
Gastric cancer *two GC cell lines + patient and adjacent normal tissues*	IFITM2	Functionally required for epithelial–mesenchymal transition (EMT; regulatory study)	Oncogenic; pro‐EMT	Silencing inhibits TGF‐β1‐mediated EMT via SMAD2/3 and downstream transcription factors	IFN‐input: not IFN‐defined (TGF‐β1/SMAD2/3) Compartment: tumour cell	Candidate therapeutic target in GC and other tumours	**C**	^25^
Pancreatic ductal adenocarcinoma *PDAC cells + co‐cultured natural killer cells*	IFITM2 *(reported as a receptor for secreted BAG3 — novel role)*	Functional study; PDAC‐secreted BAG3 (sBAG3) engages IFITM2	Oncogenic; immunosuppressive (NK)	sBAG3 binds IFITM2 → activates MAPK/phospho‐ERK; sBAG3 also diminishes NK‐cell cytotoxicity and effector‐molecule expression	IFN‐input: not IFN‐defined (sBAG3/MAPK) Compartment: tumour cell; NK‐cell (immune effector) crosstalk	sBAG3–IFITM2 axis proposed as a dual target acting on tumour cells and immune effectors	**C**	^108^
Gastric cancer *public database + 167 GC patients + in vivo lung‐metastasis model*	IFITM2	↑ in GC; positively correlated with progression, recurrence and mortality	Oncogenic; pro‐metastatic	IGF1 → IGF1R/STAT3 → IFITM2; IFITM2 in turn drives IL‐6 expression/secretion (positive feedback loop)	IFN‐input: not IFN‐defined (IGF1/STAT3/IL‐6) Compartment: tumour cell	Silencing suppresses tumour growth and lung metastasis in vivo; novel prognostic biomarker	**B**	^106^
Gastric cancer (early disease) *single‐cell transcriptomics + DNA‐methylation + CRISPR knockout across 35 GC lines + siRNA*	IFITM2	↑ already in early GC epithelial cells with promoter hypomethylation; high IFITM2 → poor prognosis	Oncogenic; initiation marker	Activates TNF‐α–NF‐κB signalling; silencing reduces NF‐κB activity and targets (BCL‐2, BCL‐XL, TNF‐α, CCL2, CDK1, COX‐2)	IFN‐input: not IFN‐defined (TNF‐α/NF‐κB) Compartment: tumour cell (epithelial)	Prognostic biomarker and therapeutic target in GC initiation and progression	**B**	^62^
Glioblastoma multiforme — elderly (≥ 60 y) *CGGA + TCGA + GSE16011 + IHC for CD8 and IFITM2 + siRNA*	IFITM2	↑ in elderly vs. younger GBM; inflammatory TME with more CD8^+^ infiltrate; shorter survival	Oncogenic; IFN‐γ‐induced (age‐related)	CD8^+^‐T‐cell‐derived IFN‐γ triggers IFITM2 expression → IFITM2 mediates the malignant phenotype induced by IFN‐γ	IFN‐input: IFN‐γ (type II), CD8‐derived (inflammatory TME) Compartment: tumour cell (induced by immune‐derived IFN‐γ)	Helps explain the inferior elderly‐GBM outcome through an age‐specific IFN‐γ/IFITM2 axis	**C**	^109^
**IFITM5, member‐specific divergence (osteosarcoma)**								
Osteosarcoma *SaOS2 cells transfected with wild‐type IFITM5 and IFITM5 c.‐14C>T mutant; Alizarin Red mineralisation assay*	IFITM5 *and c.‐14C>T variant*	Forced overexpression of wild‐type and mutant IFITM5 (mutant expressed at higher level)	Tumour‐suppressive/pro‐differentiative	Drives osteogenic differentiation: ↑ ALP, OCN, Runx2 and mineralised nodules; ↑ apoptosis; ↓ invasion (mutant additionally ↓ migration); no change in proliferation	IFN‐input: N/A (non‐IFN‐inducible member) Compartment: tumour cell (osteoblast‐like)	Suggests IFITM5 as a differentiation‐promoting therapeutic concept in osteosarcoma — directionally opposite to IFITM1/2/3 in other tumours	**C**	^111^
**IFITM10, across tumours**								
Colorectal cancer *TCGA + CCK‐8 + tube‐formation assay + IF/WB/ELISA*	IFITM10	↑ in CRC; positively correlated with tumour angiogenesis	Oncogenic; pro‐angiogenic	IFITM10 inhibition reduces STAT3 phosphorylation; IFITM10‐mediated angiogenesis is STAT3‐dependent	IFN‐input: N/A (non‐IFN‐inducible member) Compartment: tumour cell; endothelial crosstalk	Candidate prognostic biomarker and therapeutic target in CRC	**C**	^112^
Gastric cancer *TCGA + qPCR in surgical samples; ROC analysis*	IFITM10	↑ in GC vs. normal (AUC .813 overall; .955 in paired tissues). HIGHER in T1/T2 than in higher‐T‐stage tumours	Early‐diagnostic biomarker	GSEA‐suggested signalling association; mechanistic depth limited	IFN‐input: N/A (non‐IFN‐inducible member) Compartment: tumour cell	Useful for early diagnosis; expression DECREASES with advancing T stage — caveat: not a progression/poor‐prognosis marker, unlike many IFITM observations	**C**	^113^

**Axis mapping**: The four contextual axes are resolved across the table: Member identity (column ‘IFITM member’), Tumour‐milieu/context (column ‘Cancer (subtype) and evidence base’), and IFN‐input quality and Cellular compartment (column ‘Axis context’). Member‐specific IFN responsiveness is preserved: IFITM2 is an IFN‐inducible, immunity‐related member (marked ‘not IFN‐defined’ where the study did not manipulate IFN, or with the specific IFN input where it did, e.g., CD8‐derived IFN‐γ in elderly GBM), whereas *IFITM5 and IFITM10 are non‐IFN‐inducible and are therefore marked ‘N/A (non‐IFN‐inducible member)’* rather than being collapsed into IFN‐family assumptions.

**Evidence tiers**: A: Member‐specific causal evidence from in vivo functional perturbation (genetic deletion, knockdown or targeted pharmacological intervention), corroborated in immune‐competent models and/or independent human cohorts; B: In vivo or robust mechanistic evidence demonstrating a partial causal chain, including xenograft, CRISPR‐based or other mouse‐model studies, as well as single‐cohort evidence supported by functional experiments; C: In vitro, correlative, marker‐level or computational evidence lacking in vivo functional validation; D: Preliminary biomarker‐ or association‐level evidence from unvalidated single cohorts, conference abstracts or studies lacking both external validation and functional support. Conference abstracts are identified as such in the evidence‐base column and are assigned Tier D unless functional evidence is available.

**Symbols**: ↑, increased/upregulated; ↓, decreased/downregulated; →, leads to.

**TABLE 4:** Study‐level evidence for interferon‐induced transmembrane (IFITM)‐dependent regulation of antigen presentation and immune recognition.

Cancer (subtype) and evidence base	IFITM member	Expression vs. normal/control	Functional role	Key mechanism/molecular axis	Axis context (IFN‐input · compartment)	Clinical, prognostic and translational findings	Tier	Refs.
**Transcriptional control of major histocompatibility complex class I (MHC‐I; NLRC5 axis)**								
Small‐cell lung cancer (SCLC) *single‐cell and bulk RNA‐seq; multi‐cohort PD‐1/PD‐L1‐treated patients; IHC H‐score; IFITM3 induction in vitro; mouse SCLC + anti‐PD‐1*	IFITM3	↑; correlates with MHC‐I across multiple SCLC cohorts	Pro‐immunogenic; immunosensitising	IFITM3 upregulates NLRC5 and drives its nuclear translocation → MHC‐I transactivation, antigen‐presentation enrichment, ↑ CD8^+^ T‐cell infiltration and cytotoxicity	IFN‐input: not IFN‐defined (IFITM3 raised genetically or by ethyl gallate)^a^ · Compartment: tumour cell → CD8^+^ T cell	High IFITM3 → longer PFS on chemoimmunotherapy but not chemotherapy alone (predictive, not merely prognostic); IFITM3 induction sensitises mouse SCLC to anti‐PD‐1	**A**	^50^
**Wnt/β‐catenin‐coupled human leukocyte antigen class I (HLA‐I) downregulation and NK‐cell recognition**								
Triple‐negative breast cancer *isogenic MDA‐MB‐231 overexpressing and BT‐20 knockdown lines; RNA‐seq; β‐catenin reporter; LDH NK‐cytotoxicity; β‐catenin and JAK inhibitors; patient IHC*	IFITM1	Gain‐ and loss‐of‐function models; patient IHC shows a non‐significant trend only	Immune‐modulatory; effector‐dependent	IFITM1 → Wnt/β‐catenin activity → ↓ HLA class I → ↑ NK missing‐self lysis; β‐catenin or JAK inhibition restores HLA class I and reduces NK killing	IFN‐input: not IFN‐defined (no IFN manipulated; JAK inhibition only)^a^ · Compartment: tumour cell → NK cell	Opposite direction for the two effector arms: ↓ HLA class I favours NK killing but predicts impaired CD8^+^ recognition, which was not tested^b^	**C**	^27^
**Translation‐level control of HLA‐B and MHC class I (SRSF1/ISG15 axis)**								
Cervical carcinoma (SiHa) *SBP‐tagged IFITM1 affinity purification; proximity ligation; isogenic CRISPR IFITM1/IFITM3 double‐null; RNA‐seq; ribosome profiling; complementation rescue*	IFITM1 + IFITM3 (deleted together)^c^	Endogenous, IFN‐γ‐inducible; double‐null vs. parental	Translation‐level regulator of HLA‐B	IFITM1 associates with SRSF1 and, under IFN‐γ, with HLA‐B mRNA; double‐null lowers HLA‐B protein and the 80S ribosomal fraction without transcript change; rescue partially restores 80S	IFN‐input: acute/transient IFN‐γ (defined exogenous stimulus) · Compartment: tumour cell	Establishes a combined IFITM1/IFITM3 requirement for HLA‐B translation, not interchangeability^c^. In vitro only; no cohort validation	**C**	^64^
Cervical carcinoma (SiHa) *patient FFPE IHC; isogenic Cas9 IFITM1/IFITM3 double‐null; pulse‐SILAC; SWATH‐IP mass spectrometry; IFITM1 siRNA with SWATH‐MS*	IFITM1 + IFITM3 (double‐null); IFITM1 (siRNA)^c^	Endogenous; IFITM1/3 protein inversely related to metastasis in patient subgroups	Regulator of IFN‐γ‐stimulated protein synthesis	Double‐null attenuates IFN‐γ‐induced IRF1, HLA‐B and ISG15 synthesis and ISG15ylation and lowers surface HLA‐B; IFITM1 siRNA attenuates an ISG protein subset including MHC class I	IFN‐input: acute/transient IFN‐γ (defined exogenous stimulus) · Compartment: tumour cell	Links IFITM1/3 loss to reduced MHC class I output and proposes immune escape in IFITM1/3‐negative cervical cancer^d^. In vitro and correlative IHC only	**C**	^45^
**Pan‐cancer correlative and contextual evidence**								
Pan‐cancer, in depth in bladder cancer *TCGA transcriptomes; therapy‐response modelling; IMvigor210 and one recruited cohort*	IFITM3	↑ in most tumours vs. adjacent tissue	Correlative immunological biomarker	IFITM3 correlates with immunomodulators, immune infiltrate and cancer‐immunity‐cycle steps and, simultaneously, with inhibitory checkpoints; associates with an inflamed phenotype	IFN‐input: not IFN‐defined (bulk signature; quality and duration unresolved)^a^ · Compartment: bulk tumour + immune infiltrate (deconvolution)	Predicts higher response to immunotherapy, chemotherapy and anti‐EGFR therapy and lower response to anti‐ERBB2/ERBB4 and antiangiogenics. No perturbation; not a decision tool^e^	**C**	^126^
Head and neck cancer *MORC3 RNA interference with RNA‐seq and qRT‐PCR; pan‐cancer survival analysis. Not an IFITM‐functional study*	None perturbed (IFITM1, IFITM3 among ten co‐induced ISGs)	IFITM1 and IFITM3 transcripts rise after MORC3 knockdown	Context only	MORC3 knockdown upregulates PD‐L1, STAT1, an ISG set including IFITM1 and IFITM3, and the lncRNA LINC00880; LINC00880 silencing attenuates PD‐L1	IFN‐input: not IFN‐defined (MORC3‐dependent ISG de‐repression) · Compartment: tumour cell	PD‐L1 mechanism runs through LINC00880, not through any IFITM; IFITMs are neither measured at protein level nor perturbed	**—**	^127^

**Axis mapping**: Rows are grouped by regulatory layer (transcriptional control of MHC‐I; Wnt/β‐catenin‐coupled HLA class I downregulation; translation‐level control of HLA‐B; pan‐cancer correlative and contextual evidence). The member axis is the ‘IFITM member’ column, the tumour‐milieu axis the ‘Cancer (subtype) and evidence base’ column, and the IFN‐input quality and cellular compartment axes the ‘Axis context’ column.

**Evidence tiers**: A: Member‐specific causal evidence from in vivo functional perturbation (genetic deletion, knockdown or targeted pharmacological intervention), corroborated in immune‐competent models and/or independent human cohorts; B: In vivo or robust mechanistic evidence demonstrating a partial causal chain, including xenograft, CRISPR‐based or other mouse‐model studies, as well as single‐cohort evidence supported by functional experiments; C: In vitro, correlative, marker‐level or computational evidence lacking in vivo functional validation; D: Preliminary biomarker‐ or association‐level evidence from unvalidated single cohorts, conference abstracts or studies lacking both external validation and functional support. Conference abstracts are identified as such in the evidence‐base column and are assigned Tier D unless functional evidence is available. ‘—’ marks context‐only rows that do not provide IFITM‐specific functional or associative data (the study does not measure or manipulate an IFITM) and are included solely to frame the upstream IFN‐input or checkpoint context; these are deliberately not assigned an IFITM evidence tier so that different evidence types are not treated as equivalent support.

^a^ The acute‐versus‐chronic IFN input was not experimentally defined in this study; the assignment shown is inferred from the downstream antigen‐presentation readout rather than from a manipulated IFN exposure.

^b^ The opposite consequence for CD8^+^ T‐cell recognition is implied by the reduced HLA class I but was not experimentally tested.

^c^ IFITM1 and IFITM3 were deleted together, and the detection antibody recognises an epitope shared by the two paralogs; the result therefore establishes a *combined* IFITM1/IFITM3 requirement and is explicitly not evidence of functional interchangeability.

^d^ Immune escape in IFITM1/3‐negative tumours is proposed by the authors of the primary study and is not demonstrated experimentally.

^e^ Correlative only; no functional perturbation was performed, so these associations are not validated for clinical decision‐making.

**Symbols**: ↑, increased/upregulated; ↓, decreased/downregulated; →, leads to.

**TABLE 5:** Study‐level evidence for interferon‐induced transmembranes (IFITMs) in immune compartments, stroma and adaptive resistance.

Compartment/setting and evidence base	IFITM member tested	Direction of immune effect	Key mechanism/molecular axis	Axis context (IFN‐input · compartment)	Clinical/translational findings and critical caveats	Tier	Refs.
	**IFITMs across immune compartments (immune‐cell‐intrinsic compartment)**						
Tumour‐associated macrophages, lung adenocarcinoma (LUAD) *lung adenocarcinoma; mouse tail‐vein model + monocyte co‐culture + IHC in patients*	IFITM1	Pro‐tumour (inflammatory)	PLK1‐phosphorylated FoxM1 (Ser25) → STING–TBK1–IRF3 → IFITM1; IFITM1 amplifies IL‐1A/B, IL‐6, VEGFA → M2d‐like TAM polarisation and PD‐L1 upregulation	IFN‐input: type I IFN (STING–TBK1–IRF3‐driven, pro‐inflammatory) Compartment: tumour‐associated macrophage (myeloid)	Higher FOXM1/PLK1/IFITM1 inversely correlates with survival in advanced LUAD; supports a FoxM1–IFITM1 therapeutic axis against immune escape	**B**	^128^
Engineered‐macrophage adoptive therapy, melanoma *mouse melanoma + clinical metastatic samples; hyperthermia rescue*	IFITM1 (reported as an M1‐macrophage‐associated ISG; not functionally manipulated)	Context only (IFITM1 reported at expression level; no IFITM loss‐ or gain‐of‐function evidence)	CD133^+^PD‐L1^+^ subpopulation builds a TGF‐β‐dependent stiff‐ECM niche that excludes transferred macrophages and suppresses their cell‐killing; hyperthermia restores the calreticulin ‘eat‐me’ signal. IFITM1 is reported among interferon‐stimulated genes expressed by M1‐polarised macrophages in this system	IFN‐input: IFN‐conditioned M1 macrophage program (IFITM1 as ISG readout); tumour niche not IFITM‐defined (TGF‐β/ECM) Compartment: myeloid (adoptive/M1‐polarised macrophages); tumour ECM niche	IFITM1 is reported as an M1‐associated ISG but is not perturbed, so this study identifies IFITM1 as a myeloid‐compartment marker rather than demonstrating IFITM1‐dependent resistance. Cited as parallel evidence of checkpoint‐linked resistance to myeloid therapy; the causal IFITM link is interpretive and not provided by the primary data	**C**	^129^
Splenic, thioglycollate‐elicited and bone‐marrow‐derived macrophages, tumour‐bearing mice *comparison of IFITM family expression; LPS/TLR stimulation (comparative IFITM1‐5/6 screen)*	IFITM6 (mouse‐specific paralog; also comparative IFITM1‐5 screen)	Pro‐tumour (inflammatory; marker‐level)	IFITM6 induced in macrophages but not in T cells; LPS and TLR1, 2, 3, 4 and 9 agonists raise IFITM6 expression	IFN‐input: TLR/LPS‐induced (innate; ISG‐adjacent) Compartment: macrophage (myeloid)	Expression‐only observation; no functional manipulation — implicates, but does not demonstrate, a role for IFITM6 in tumour‐bearing‐host macrophage biology	**C**	^130^
IBD‐associated colorectal cancer *GSE165512 (n = 170) + machine learning + DSS/anti‐CD40 mouse colitis + cBioPortal*	IFITM1 (one of nine SHAP‐ranked genes; AQP9, CXCL8, ITGA5, others)	Pro‐tumour (inflammatory; computational)	Computational signature links IFITM1 to epithelial–mesenchymal transition (EMT), PI3K/AKT signalling and immune‐cell infiltration in IBD‐CRC; RT‐PCR confirms overexpression in DSS colitis (*p* < .0001)	IFN‐input: not IFN‐defined (acute DSS colitis; computational) Compartment: tumour + immune infiltrate (bulk/computational)	Best diagnostic AUCs were for ITGA5 and CXCL8, NOT IFITM1; IFITM1 is one candidate among several and requires direct functional validation in IBD‐CRC	**C**	^131^
Tumour‐infiltrating FOXP3^+^ regulatory T cells *in vivo Treg‐specific perturbation; multiple syngeneic tumour models. Strongest immune‐cell‐intrinsic anchor*	IFITM3	Pro‐immunogenic (when IFITM3 is lost)	IFNγ → STAT1 → IFITM3 forms a feedback loop maintaining Treg fragility. IFITM3 deletion enhances STAT1 translation/phosphorylation → Th1‐like, IFNγ‐secreting Treg phenotype → anti‐tumour immunity	IFN‐input: IFN‐γ (type II)–STAT1 feedback Compartment: regulatory T cells (Tregs; lymphoid)	Identifies the IFN‐γ–STAT1–IFITM3 loop as a targetable Treg vulnerability for cancer immunotherapy. This Treg‐intrinsic mechanism is fully compatible with IFN‐γ increasing major histocompatibility complex class I (MHC‐I) in tumour cells: the two effects act in different compartment	**A**	^65^
B cells, normal and B‐cell leukaemia/lymphoma *clinical cohorts + Ifitm3^−^/^−^ mice + phosphomimetic IFITM3(Y20E)*	IFITM3	Pro‐tumour (B‐cell oncogenic scaffold)	BCR engagement/oncogenic kinases phosphorylate IFITM3 at Tyr20 → surface accumulation; IFITM3 acts as a PIP3 scaffold via Lys83/Lys104 to amplify PI3K signalling; required for germinal‐centre formation and for oncogene‐driven B‐cell transformation	IFN‐input: not IFN‐defined (BCR/PIP3 scaffold; IFN‐independent function) Compartment: B cell (lymphoid)	Strong predictor of poor outcome in B‐cell leukaemia and lymphoma cohorts; defines a druggable PIP3‐scaffold function distinct from the endosomal antiviral role	**A**	^58^
NK cells, dendritic cells and macrophages *Irf3^−^/^−^ mice + B16 melanoma + poly‐I:C therapy. Caveat: study is about IRF3, not IFITMs*	IRF3 (upstream of IFN/ISG axis; not IFITM‐specific)	Context only (upstream IFN‐axis, not IFITM)	IRF3 controls poly‐I:C‐induced NK Granzyme B and IFN‐γ, dendritic‐cell TIM‐3 reduction, macrophage IL‐12/IL‐15/IL‐6 and INAM	IFN‐input: upstream type I IFN axis (poly‐I:C/IRF3) Compartment: NK, dendritic cell, macrophage (innate)	Supports the upstream IFN‐quality axis but does NOT demonstrate IFITM function. Included only to frame the IFN‐input that IFITMs respond to	**—**	^133^
Bone‐marrow mononuclear cells, CAR‐T‐treated B‐ALL *single‐cell + bulk RNA‐seq in 32 patients; CAR‐T/Nalm‐6/THP1 co‐culture*	IFITM1	Pro‐tumour (inflammatory; cytokine‐release‐syndrome correlate)	IFITM1 emerges as a hub gene in IFN‐related signalling enriched in high‐grade cytokine release syndrome (CRS); in vitro knockdown reduces IL‐6 in Nalm‐6/THP1 – CAR‐T co‐culture	IFN‐input: IFN‐related signalling (ISG; acute CRS context) Compartment: bone‐marrow mononuclear/myeloid (innate)	Candidate early biomarker for severe CRS; small cohort (*n* = 28 for bulk validation) and single‐centre	**C**	^134^
Prostate adenocarcinoma, immune‐infiltrate correlation *GSE32571/GEO bioinformatics only*	IFITM1	Tumour‐suppressive correlation (in silico)	GSEA enrichment in Th1/Th2/Th17 differentiation, NK cytotoxicity, DC activation; correlates with 22 tumour‐infiltrating immune‐cell types	IFN‐input: not IFN‐defined (in silico immune correlation) Compartment: tumour + immune infiltrate (in silico deconvolution)	LOW IFITM1 → poor prognosis in PRAD, directionally opposite to the dominant IFITM1 pattern in epithelial tumours. Caveat: bioinformatics only, no functional validation	**C**	^135^
Brain‐metastasis surveillance *in vivo genome‐wide CRISPR–Cas9 screen in lung‐cancer brain‐metastasis model + clinical ICB data. Directionally opposite to most IFITM1 findings: surveillance, not progression*	IFITM1	Pro‐immunogenic (when retained on tumour cells)	LOSS of IFITM1 in lung‐cancer cells promotes brain colonisation. Intact tumour‐cell IFITM1 secretes complement C3 → microglial activation, and elevates surface MHC‐I → enhanced CD8^+^ T‐cell killing	IFN‐input: not IFN‐defined (C3–microglia–MHC‐I surveillance) Compartment: tumour cell; microglia/CD8^+^ T‐cell crosstalk	IFITM1 expression correlates with ICB response; combining IFITM1 induction with anti‐PD‐1 reduces brain metastasis in mice	**B**	^59^
	**Stroma, MSCs, microbiome and single‐cell TME (stromal/microbiota compartment, IFN‐input conditioning)**						
Cancer‐associated fibroblasts, breast cancer *primary human CAF cultures + MCF‐7 indirect co‐culture + independent microarray validation*	Type I IFN response in a CAF subgroup (IFITM not directly measured)	Context only (IFN‐input conditioning; pro‐tumour)	A CAF subgroup is positive for a type I IFN response and promotes MCF‐7 proliferation in an IFN‐β‐dependent manner; neutralising IFN‐β reverts the effect	IFN‐input: type I IFN (IFN‐β), CAF‐derived (chronic/stromal) Compartment: cancer‐associated fibroblast (stromal) → tumour	‘Interferon‐positive’ CAF state is associated with poor prognosis in an independent cohort. In vitro evidence only; not validated in vivo. Defines a stromal source of IFN that downstream tumour IFITMs would respond to	**—**	^136^
Adipose‐tissue‐derived MSCs + radiotherapy, HCC *direct and indirect co‐culture + xenograft*	IFITM1 (reported as part of an RNA‐seq signature)	Anti‐tumour (treatment‐enhancing context)	AT‐MSCs enhance RT efficacy with an IFITM1‐induced gene signature; downstream effects include STAT3 and MMP downregulation and p53/caspase upregulation	IFN‐input: not IFN‐defined (radiotherapy‐induced signature) Compartment: mesenchymal stem cell (stromal) → tumour	Preclinical rationale for combining AT‐MSCs with RT in HCC. Mechanistic linkage to IFITM1 is associative, not causal	**C**	^137^
Gut microbiota, clear‐cell renal cell carcinoma *patient faecal microbiome + mechanistic studies + biofilm‐coated probiotic*	IFITM1 (downstream of HOXD10)	Anti‐tumour (microbiota‐conditioned)	Lachnospiraceae‐derived propionate (SCFA) downregulates HOXD10 and its downstream IFITM1 → activates JAK1–STAT1/2 signalling → inhibits ccRCC proliferation and migration	IFN‐input: SCFA‐conditioned; JAK1–STAT1/2 (IFN signalling) Compartment: gut microbiota (host‐microbial interface) → tumour	Single mechanistic report; ‘ccRCC IFITM1 ↑ = bad’ in ccRCC is consistent with Section 3; the microbiota intervention adds a new translational angle but rests on one cohort	**B**	^138^
Gut epithelium and immunity, colitis and colitis‐associated tumourigenesis *IFITM3‐deficient mice + whole IFITM‐locus‐deficient mice + 16S rRNA microbiota analysis + bone‐marrow transplantation. Directionally opposite to most tumour‐cell‐intrinsic IFITM3 findings*	IFITM3 (with whole IFITM‐locus deletion models; member‐specific attribution limited). See Section 2.4 for why this phenotype cannot be attributed to an individual member.	Tumour‐suppressive/protective in colitis‐associated cancer	IFITM3/whole‐locus deletion → worse DSS‐induced colitis, skewing towards pathogenic Th17 with reduced IL‐10, altered microbiota and increased invasive tumourigenesis; haematopoietic‐cell‐derived effect (BMT)	IFN‐input: not IFN‐defined (DSS colitis/microbiota; whole‐locus) Compartment: gut epithelium + haematopoietic immune (host)	In this organ/inflammation context IFITM3 is anti‐inflammatory and partially protective — a context‐specific divergence that must be flagged when generalising IFITM3 biology across tumours	**B**	^63^
Stromal and immune subtypes, gastric tumour microenvironment *single‐cell RNA‐seq + NMF + SCENIC + IHC validation*	IFITM1 (IFITM1+ CAF‐C3 subtype)	Prognostic stratification (TME context)	Oncofetal reprogramming defines an IFITM1+ CAF‐C3 subtype alongside other CAF, TEC and TAM subtypes; transcription‐factor networks differ across subtypes	IFN‐input: not IFN‐defined (single‐cell descriptive) Compartment: stromal CAF‐C3 (single‐cell TME)	IFITM1+ CAF‐C3 is one of several prognostic and ICB‐predictive subtypes, not an IFITM‐functional study; supports a descriptive role rather than causation	**C**	^26^
	**IFITMs, immune checkpoints and adaptive immune resistance (checkpoint/adaptive‐resistance compartment)**						
Triple‐negative breast cancer *bioinformatics + clinical validation + xenograft + anti‐PD‐1 in vivo. Tier A anchor for adaptive resistance*	IFITM3	Adaptive resistance (via PD‐L1)	Enriched H3K27ac at the IFITM3 promoter → IFITM3 transcription → TNF‐α–NF‐κB activation → PD‐L1 upregulation; blocking TNF‐α/NF‐κB reverses the pro‐cancer effect	IFN‐input: chronic inflammatory (H3K27ac–TNF‐α/NF‐κB‐coupled) Compartment: tumour cell (→ CD8^+^ T‐cell exclusion)	IFITM3 overexpression confers resistance to anti‐PD‐1; knockdown restores sensitivity, prolongs survival, increases CD8^+^ infiltration	**A**	^61^
Gastric cancer *TCGA + GEO + IHC + MKN28/MKN45 + Jurkat T‐cell co‐culture + xenograft*	IFITM2	Adaptive resistance (via PD‐L1)	Tumour‐derived IFITM2 activates JAK/STAT3 → induces PD‐L1 → drives Jurkat T‐cell apoptosis in co‐culture and in xenografts	IFN‐input: chronic (JAK/STAT3–PD‐L1‐coupled) Compartment: tumour cell (→ T‐cell apoptosis)	IFITM2 expression correlates with lymphatic metastasis, advanced stage and poor survival; therapeutic target for GC immunotherapy	**B**	^122^
Tumour‐intrinsic mitochondrial regulator, cancer‐stem‐cell populations *in vitro + in vivo PD‐L1 blockade. Caveat: not an IFITM‐functional study — included for IFN‐quality/PD‐L1 framing*	SLC25A1 → cGAS–STAT1 IFN‐I + PD‐L1 (IFITMs not directly manipulated)	Context only (dual: pro‐immunogenic IFN‐I + adaptive resistance)	SLC25A1 drives mitochondrial‐DNA cytosolic accumulation → cGAS–STAT1 IFN‐I (virus‐mimicry state); separately stabilises PD‐L1 via a fumarate–Keap1 axis	IFN‐input: type I IFN (cGAS–STAT1, virus‐mimicry) Compartment: tumour cell/cancer stem cell	High SLC25A1 → inflamed TME + paradoxical sensitivity to anti‐PD‐L1. Useful for the IFN‐quality argument but not direct IFITM evidence	**—**	^139^
Prostate cancer after androgen‐signalling inhibition *conference abstract (AACR Annual Meeting 2026; not peer‐reviewed); spatial + single‐cell + bulk transcriptomics; 124 patients + matched pre‐/post‐treatment + 10‐patient spatial. Caveat: IFITM signal is secondary, not the study's focus*	IFITM1, IFITM2, IFITM3 (within a broader ISG/MHC‐I/II signature)	Therapy‐resistance phenotype (descriptive)	Androgen‐deprivation therapy + androgen‐receptor signalling inhibitor promote a mixed KRT5+/TP63+ basal and PIGR+/KLF5+ club‐like cell identity with upregulated MHC class I/II and IFITM1/2/3; pro‐inflammatory chemokine signalling induces ISG features in vitro	IFN‐input: ISG/MHC program (IFN‐associated; therapy‐induced) Compartment: tumour cell (basal/club‐like identity)	State associated with reduced AR‐targeted‐therapy sensitivity; the IFITM signal is embedded in a broader immunomodulatory program and should not be cited as an IFITM‐focused study	**D**	^140^

**Axis mapping**: Rows are grouped by the three host compartments (immune‐cell‐intrinsic; stromal/microbiota; checkpoint/adaptive‐resistance); the per‐row ‘Axis context’ column states the IFN‐input quality and the precise cellular compartment. The member axis is the ‘IFITM member tested’ column and the tumour‐milieu axis is the setting column.

**Evidence tiers**: A: Member‐specific causal evidence from in vivo functional perturbation (genetic deletion, knockdown or targeted pharmacological intervention), corroborated in immune‐competent models and/or independent human cohorts; B: In vivo or robust mechanistic evidence demonstrating a partial causal chain, including xenograft, CRISPR‐based or other mouse‐model studies, as well as single‐cohort evidence supported by functional experiments; C: In vitro, correlative, marker‐level or computational evidence lacking in vivo functional validation; D: Preliminary biomarker‐ or association‐level evidence from unvalidated single cohorts, conference abstracts or studies lacking both external validation and functional support. Conference abstracts are identified as such in the evidence‐base column and are assigned Tier D unless functional evidence is available. ‘—’ marks context‐only rows that do not provide IFITM‐specific functional or associative data (the study does not name, measure or manipulate an IFITM) and are included solely to frame the upstream IFN‐input or checkpoint context; these are deliberately not assigned an IFITM evidence tier so that different evidence types are not treated as equivalent support. Preprints are identified as such in the evidence‐base column and are treated as provisional evidence.

**Symbols**: ↑, increased/upregulated; ↓, decreased/downregulated; →, leads to.

*Evidence grading*. For this structured narrative review, we applied an author‐defined operational evidence‐tier framework to the included findings. The tiers were used consistently throughout the text and in the ‘Tier’ column of Tables 1, 2, 3, 4, 5. **Tier A** comprised member‐specific causal evidence derived from in vivo functional perturbation, such as genetic deletion, knockdown or targeted pharmacological intervention, with corroboration in immune‐competent models and/or independent human cohorts. **Tier B** included in vivo or robust mechanistic evidence demonstrating a partial causal chain, including xenograft, CRISPR‐based or other mouse‐model studies, as well as single‐cohort evidence supported by functional experiments. **Tier C** encompassed in vitro, correlative, marker‐level or computational evidence lacking in vivo functional validation. **Tier D** was reserved for preliminary biomarker‐ or association‐level evidence derived from unvalidated single cohorts, conference abstracts or studies lacking both external validation and functional support.

### 2.1 Structural and functional diversity of the IFITM paralogs

IFITMs are small membrane proteins of the dispanin/CD225 superfamily. Although this superfamily is ancient and includes prokaryotic members, the IFITM proteins themselves are confined to eukaryotes, and the immunity‐related members are vertebrate‐specific.^6^, ^29^ Vertebrate IFITM family is composed of three subfamilies with distinct structural and functional characteristics, suggesting that each of them derived from a different ancestor: an immune‐related IFITM subfamily (including IFITM1, IFITM2 and IFITM3), IFITM5 and IFITM10.^6^ The IFITM protein family is crucial in the immune defense,^5^, ^30^ especially in the engagement of virus^31^, ^32^, ^33^ and in regulating tumourigenesis.^12^, ^28^ Human IFITM3 is thought to have the broadest and most potent inhibitory activity against a range of viruses, followed by IFITM1 and IFITM2, which show narrower and generally weaker restriction profiles. Taken together with phylogenetic evidence, IFITM3 was likely to be the earliest antiviral IFITM family member and subsequent duplications and speciations gave rise to the other immune‐related IFITMs.^6^, ^34^ Each of the IFITM1, IFITM2 and IFITM3 genes is an ISG and harbours an interferon‐stimulated response element (ISRE) in its promoter region, in addition to an extra gamma‐activated sequence (GAS) present in the promoter region of the IFITM1 gene.^29^ However, IFITM5 and IFITM10 are not IFN‐inducible, and no antiviral activity has been established for either member in mammalian systems, although characterisation of an IFITM10 orthologue in chicken has been reported.^8^, ^9^

**FIGURE 1:**
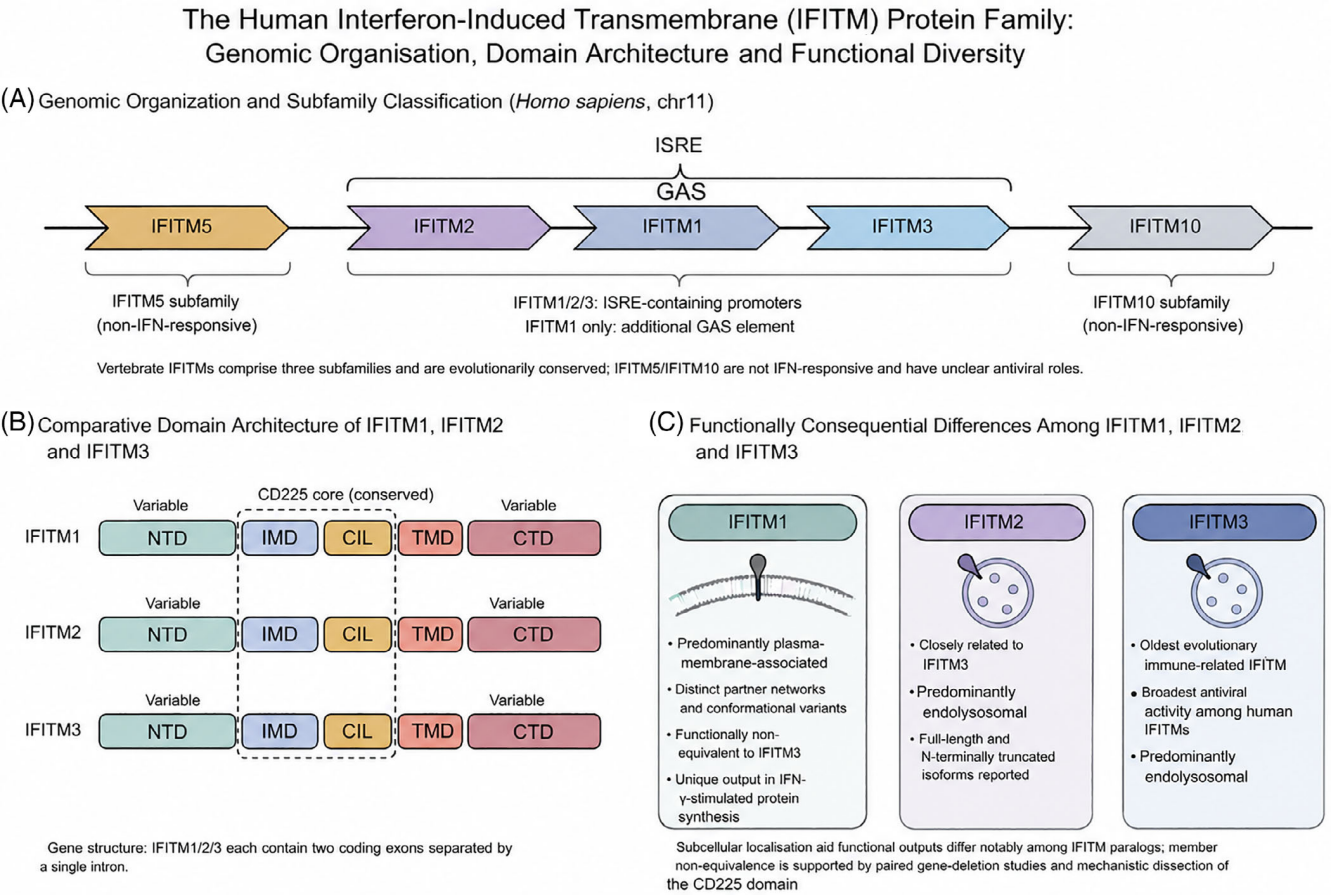
Genomic organisation, domain architecture and functional diversity of the human IFITM paralogs. (A) Human interferon‐induced transmembranes (IFITMs) on chromosome 11 are organised into three subfamilies: the immune‐related IFITMs, IFITM1/2/3; IFITM5; and IFITM10. IFITM1/2/3 are IFN‐responsive IFN‐stimulated genes (ISGs) with interferon‐stimulated response element (ISRE)‐containing promoters, while IFITM1 also contains an additional gamma‐activated sequence (GAS) element. IFITM5 and IFITM10 are not interferon (IFN)‐inducible, and no antiviral activity has been established for either member in mammalian systems. (B) IFITM1, IFITM2 and IFITM3 share a conserved CD225 core spanning the intramembrane domain (IMD)–conserved intracellular loop (CIL) region, whereas N‐ and C‐terminal domains (NTD and CTD) are more variable. IFITM2/3 may also generate full‐length and N‐terminally truncated isoforms. (C) IFITM paralogs are structurally related but functionally non‐interchangeable. IFITM1 is mainly plasma‐membrane‐associated, whereas IFITM2 and IFITM3 are predominantly endolysosomal. IFITM3 shows the broadest antiviral activity among human IFITMs, while paired deletion studies support non‐overlapping IFITM1 and IFITM3 functional outputs. IFITM5 is osteoblast‐restricted and functions in mineralisation rather than in antiviral or interferon‐driven programs, and IFITM10 remains functionally uncharacterised in mammalian systems; neither is interferon‐inducible, and neither is placed on the acute‐versus‐chronic IFN axis used throughout this review.

The IFITM1, IFITM2 and IFITM3 gene sequences are closely related, with the three sharing more than 90% identity along 70% of the coding sequence. IFITM2 and IFITM3 share substantial similarity (91%), also within non‐coding regions (with the exception of a 68‐bp tandem duplication situated within the 3′ flanking region of IFITM2). In the non‐coding region, however, identity between IFITM1 and the other two genes is only 65%. All three sequences consist of two exons with a single intron in the same position.^35^ As shown in Figure 1, IFITM1, IFITM2 and IFITM3 each contain two coding exons separated by a single intron. IFITM2 and IFITM3 are predicted to encode a full‐length form and a truncated isoform with 20/21 amino acid residues from N‐terminus deletion. Most vertebrate animals possess two or more IFITM genes. In several primate species, the expansion through gene duplication, and subsequent divergence, of IFITM3 has been noted.^6^, ^34^ Comparative genomics further indicates that IFITM2 arose much more recently, as a distinct genetic event in the human–chimpanzee–gorilla lineage.^34^ The presence of multiple and diverse IFITM2/3 suggests that there is a positive selection for IFITM in evolution, which fits well with their involvement in limiting pathogen invasion.^34^ IFITMs consist of intramembrane domain (IMD) and transmembrane (TMD) domains separated by a conserved intracellular loop (CIL) and variable N‐ and C‐terminal domains (NTD and CTD), respectively.^36^, ^37^, ^38^ While the NTD and CTD are highly variable in length and sequence among IFITM orthologs and paralogs, the canonical CD225 domain spanning the IMD to CIL domains is evolutionarily more conserved (Figure 1).^37^

IFITM1, IFITM2 and IFITM3 share a conserved CD225/dispanin architecture, including intramembrane regions and a CIL, yet they differ in subcellular localisation in ways that shape their biological functions: IFITM1 is enriched at the plasma membrane/cell surface, whereas IFITM2 and IFITM3 preferentially associate with endosomal and endolysosomal compartments.^39^, ^40^, ^41^ Functionally, amphipathic features within IFITM3 contribute to membrane remodelling and antiviral fusion restriction,^38^, ^42^ while independent chemical‐genetic evidence shows that IFITM proteins can facilitate the cellular uptake of diverse linked chemotypes.^43^ Together, these findings support the view of IFITMs as active membrane‐associated effectors rather than passive scaffolding proteins.^38^, ^39^, ^42^, ^43^ Biochemical characterisation of recombinant IFITM1 has identified distinct stable conformational/oligomeric species, providing a structural basis for further investigating how IFITM1 organisation may contribute to its antiviral and cancer‐associated, including pro‐metastatic, functions.^44^ A particularly strong line of evidence for IFITM member non‐equivalence comes from CRISPR‐associated protein 9 (CRISPR/Cas9)‐based deletion studies in cervical cancer cells showing that IFITM1/IFITM3 double‐null cells showed selective attenuation of IFN‐γ‐induced synthesis of IRF1, HLA‐B and ISG15 in pulse‐SILAC analyses, whereas IFITM1 knockdown by siRNA caused broader attenuation of IFN‐regulated proteins, including MHC class I molecules, IFITM3, STAT1, B2M and ISG15. These findings argue against treating IFITM proteins as uniformly interchangeable components of the IFN response.^45^ Member‐resolved antiviral comparisons exist for several viruses; here we cite only the comparative hepatitis C virus (HCV)‐entry study by Narayana et al. as a scoped example, which directly examined IFITM1, IFITM2 and IFITM3 and showed that all three restrict HCV entry through localisation‐ and post‐translational–modification‐dependent mechanisms, illustrating functional heterogeneity rather than interchangeability among IFITM family members.^10^

The roles of IFITM family members in cancer are complex and involve several mechanisms, including participation in important signalling pathways, like JAK/STAT3,^46^, ^47^, ^48^, ^49^ MAPK^47^ and NF‐κB.^48^ However, activation or inhibition of these pathways have different outcomes, depending on the specific IFITM paralog, on tumour growth, metastasis and therapy resistance. For example, the absence of IFITM1 in MCF‐7:5C cells significantly enhanced p21 transcription, expression and nuclear localisation and this was mediated by JAK/STAT activation. Because the anti‐proliferative effect follows IFITM1 loss, IFITM1 acts as a tumour‐supportive node in this setting and is a candidate target in endocrine‐resistant breast cancer. It does not follow that IFITM1 is overexpressed across breast cancer generally.^49^ On the other hand, Provance et al. reported that IFITM1 neutralisation reduced breast cancer cell proliferation via NF‐κB.^48^ Also, in the tumour immunology regulation of IFITMs, these family members influence immune cell function. They regulate the expression and stability of MHC‐I molecules.^27^, ^50^ Because MHC‐I presents antigen to CD8‐positive (CD8^+^) T cells but engages inhibitory receptors on natural killer (NK) cells, IFN‐driven changes in MHC‐I have opposing consequences for the two compartments: higher MHC‐I favours T‐cell recognition while suppressing NK missing‐self killing, whereas MHC‐I loss has the reverse effect. IFITM family members, by tuning MHC‐I output, can therefore shift the balance between T‐cell‐ and NK‐cell‐mediated recognition of tumour cells.

### 2.2 Translational, post‐translational and upstream regulators

Translation of IFITM messenger RNAs (mRNAs) is itself gated. G3BP1 and G3BP2 regulate IFITM1/IFITM2/IFITM3 protein output in MCF7 breast cancer cells, providing one mechanism by which tumour cells can adjust IFITM abundance independently of transcription.^51^ Post‐translational state matters independently: depalmitoylation by ABHD16A reverses palmitoylation of IFITM1 and modulates its function, indicating that the active pool of IFITM protein is set by a palmitoylation cycle rather than by transcript level alone.^52^ Upstream, Matrin3 promotes IFITM1 expression in a mechanism originally characterised in hepatitis B virus biology, whose direct relevance to tumour settings remains to be established.^53^ Tumour‐cell‐intrinsic modifiers of the IFN response itself further shape IFITM output: p53 exerts complex, partially counterintuitive control over the IFN signalling system, including ISG induction^54^; cell‐line heterogeneity of IFN responses in lung adenocarcinoma (LUAD) shows that even within one tumour type, IFITM induction is not uniform across genetic backgrounds^55^; and Lipocalin‐2 regulates ISG expression in prostate cancer with downstream consequences for oncolytic virus susceptibility, illustrating tumour‐cell‐intrinsic conditioning of the IFN/IFITM axis.^56^ Collectively, these regulators imply that IFITM abundance and activity in any tumour reflect not only IFN input but also the tumour cell's own regulatory state.

### 2.3 The four‐axis framework

**FIGURE 2:**
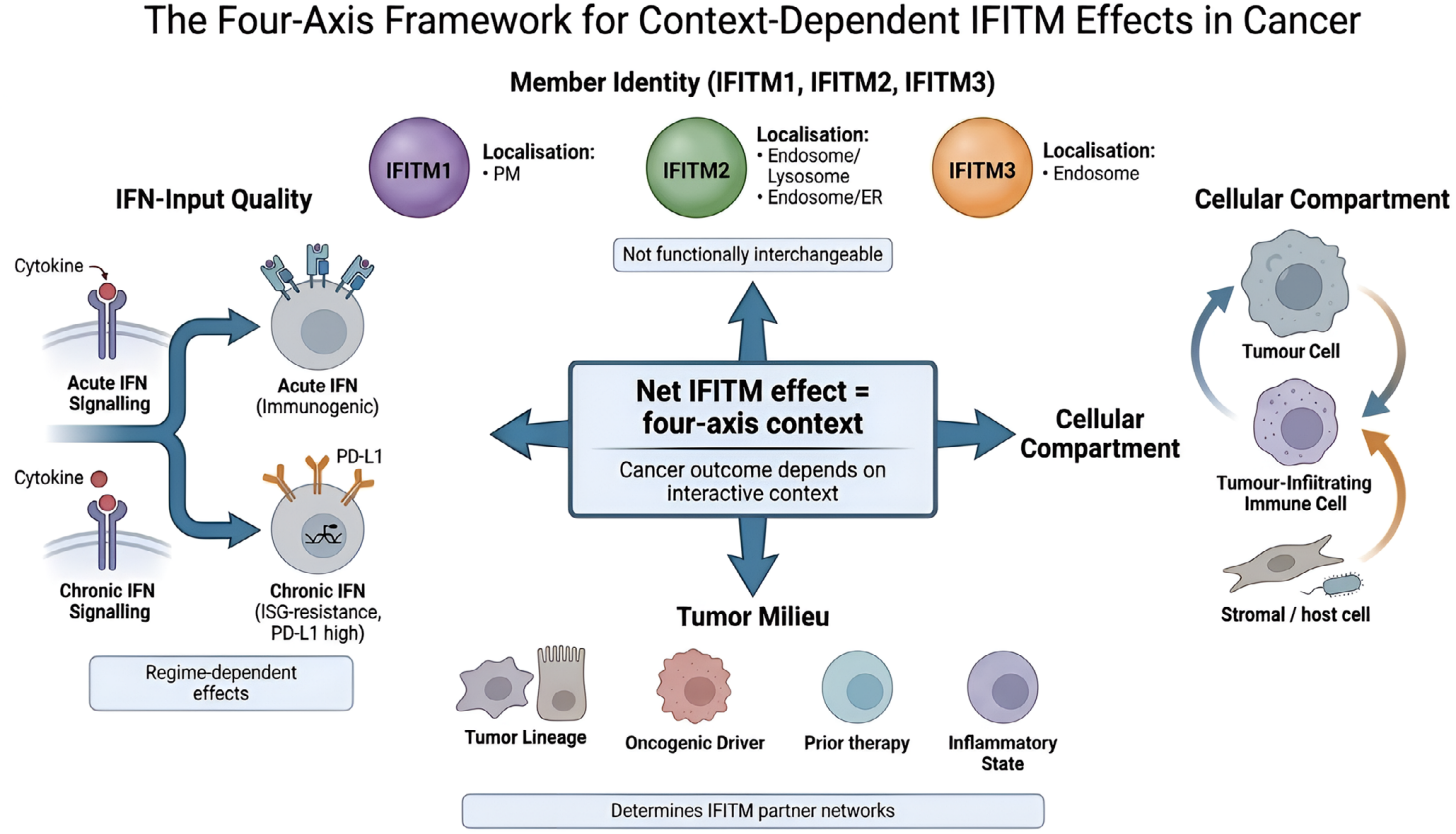
The four‐axis framework for context‐dependent interferon‐induced transmembrane (IFITM) effects in cancer. Net IFITM effect is determined by the interactive combination of four axes: member identity (IFITM1/2/3 with distinct localisation and non‐overlapping partner networks), interferon (IFN)‐input quality (acute immunogenic major histocompatibility complex class I [MHC‐I] induction versus chronic programmed death‐ligand 1 [PD‐L1]‐driven resistance), cellular compartment (tumour cell vs. tumour‐infiltrating immune or stromal host cells) and tumour milieu (lineage, oncogenic driver, prior treatment and inflammatory state). The same IFITM protein can exert opposing effects when any axis is varied. Axes interact; apparent contradictions resolve only when multiple axes are specified simultaneously (evidence largely in vitro; in vivo multi‐axis validation needed).

Sections 2.1 and 2.2 establish two constraints: the immunity‐related IFITM members are not functionally interchangeable, and their abundance and activity are set jointly by upstream IFN input and by tumour‐cell‐intrinsic regulators. The evidence in Sections 2.1, 2.4, 3.2, 4.2, 5.1, 5.2 therefore cannot be ordered along a single dimension. We instead classify each finding by four variables (‘axes’), summarised in Figure 2. A study can be scored on each axis independently; the sign and magnitude of a given IFITM effect, however, depend on the combination.

*Axis 1—Member identity (IFITM1/IFITM2/IFITM3; IFITM5 and IFITM10 where relevant)*. Members differ in subcellular localisation (Section 2.1), gene‐deletion phenotype and partner network (Sections 2.1, 2.4, 3.2), and these partner networks are largely non‐overlapping. Claims pitched at the level of ‘the IFITM family’ are not supported by this biology and are avoided throughout.

*Axis 2—IFN‐input quality (acute/transient vs. chronic/sustained)*. Acute, transient IFN signalling drives MHC class I induction and antigen presentation, the immunogenic phenotype (Section 7). Chronic, sustained IFN signalling, whether driven by persistent inflammatory signalling in the TME, by stromal‐ or microbiota‐derived IFN, or by constitutive tumour‐cell‐intrinsic ISG expression, instead selects for ISG‐resistance signatures and upregulates PD‐L1, contributing to the adaptive immune resistance examined in Section 8.3. Tonic IFN signalling, the constitutive, low‐amplitude type I IFN that maintains baseline ISG tone in healthy, steady‐state tissue,^57^ is distinguished here from chronic inflammatory signalling: tonic input sets the physiological baseline against which both acute and chronic tumour‐associated inputs are measured, and is not itself a tumour‐associated state. Studies are therefore scored on this axis relative to that baseline, and tonic signalling is not assigned to either pole.

*Axis 3—Cellular compartment (transformed tumour cell/tumour‐infiltrating immune cell/stromal or microbiota‐conditioned host cell)*. Expression in different compartments can carry opposite signs at the same anatomical site, as the colitis‐associated colorectal cancer (CRC) conflict illustrates (Section 9).

*Axis 4—Tumour milieu (tissue lineage, oncogenic driver, prior treatment exposure, inflammatory state)*. These determine which IFITM partner network is engaged, as the contrast between cervical squamous cell carcinoma and other epithelial cancers illustrates (Section 9).

Two caveats apply. First, the mechanistic studies underpinning Axes 1 and 2 (Sections 2.1 and 2.2) derive largely from in vitro and non‐tumour systems; immune‐competent in vivo dissection in a tumour context is currently lacking and is flagged as a priority evidence gap (Section 10.3). Second, the axes interact rather than add: a finding is interpretable only when at least the member, the IFN regime and the compartment are specified, which is why apparent contradictions in this literature resolve only once more than one axis is fixed (Section 9). To keep these axes visible at the point of use, Sections 3.1–8.3 each open with a short ‘Coordinates for this section’ line stating the member, IFN‐input quality, compartment, tumour milieu and the highest evidence tier available in that section. Sections 8, 8.1, 10 are synthesis rather than primary‐evidence sections and deliberately carry no coordinates line, because they draw on all four axes simultaneously rather than fixing them.

### 2.4 Evidence from IFITM gene knockout in cells and animals

The axes defined above are only as reliable as the evidence that assigns a phenotype to a specific IFITM member in a specific cell type. Because the immunity‐related paralogs share more than 90% coding‐sequence identity, are frequently co‐expressed, and are recognised by antibodies raised against shared epitopes, member attribution cannot be established by expression correlation or by partial depletion alone. Genetic ablation is therefore the decisive evidence class, and its current distribution across the field, dense in tumour cells and sparse in host compartments, is itself a structural feature of the literature.

Member‐specific knockout establishes what knockdown cannot. RNA interference leaves a residual protein pool of variable and often unquantified size, so a negative result cannot distinguish dispensability from incomplete depletion, and a positive result cannot exclude a contribution from the remaining protein; the problem is compounded here because the paralog cross‐reactivity of most IFITM antibodies makes protein‐level confirmation of knockdown efficiency unreliable. Complete genetic deletion removes this ambiguity and additionally permits rescue with defined alleles, which separates a requirement for the protein from a requirement for a particular post‐translational state. The clearest example is IFITM3 in B cells, where germline *Ifitm3*‐deficient mice combined with a phosphomimetic IFITM3(Y20E) knock‐in allele established that Tyr20 phosphorylation‐driven surface accumulation and lysine‐dependent PIP3 scaffolding, rather than IFITM3 abundance as such, are what amplify PI3K signalling and support oncogene‐driven B‐cell transformation.^58^ A comparable gain in resolution is provided by CRISPR/Cas9‐mediated deletion of IFITM1/IFITM3 in cervical carcinoma cells, in which IFN‐γ‐stimulated synthesis of selected IFN‐responsive proteins, including HLA‐B, ISG15 and IRF1, is attenuated. Complementary IFITM1‐targeted siRNA analyses further demonstrated broader attenuation of IFN‐regulated proteins, including MHC class I molecules, IFITM3, STAT1, B2M and ISG15, indicating that IFITM1/3 perturbation selectively reshapes rather than uniformly abolishes IFN‐responsive protein synthesis.^45^ In vivo genome‐wide CRISPR screening extends the same logic to phenotypes that no candidate‐based approach would have nominated, identifying tumour‐cell IFITM1 as a suppressor of brain colonisation acting through complement C3‐dependent microglial activation and surface MHC‐I elevation.^59^ By contrast, the large body of shRNA and siRNA depletion work in xenograft models, which supports the IFITM3–KLF4 axis in colon cancer,^15^ IFITM3–TRAF6/MAPK/AP‐1 in tyrosine‐kinase‐inhibitor‐resistant renal carcinoma,^47^ IFN‐α/NF‐κB‐sustained IFITM1 in triple‐negative breast cancer (TNBC),^48^ IFITM3–MET/AKT in osimertinib‐resistant LUAD,^60^ and H3K27ac‐driven IFITM3–PD‐L1 in TNBC,^61^ is mechanistically informative but is conducted almost entirely in immunodeficient hosts, so it constrains tumour‐cell‐intrinsic function without addressing the immune compartment. Array‐format CRISPR knockout across a panel of gastric cancer lines adds a further dimension by testing member‐specific dependency against genetic background rather than in a single model.^62^

Whole‐locus deletion resolves compartment but not member. The murine *Ifitm* genes are clustered, and mice carrying a deletion of the entire locus lose the immunity‐related paralogs together. In the colitis and colitis‐associated tumourigenesis model, whole‐locus‐deficient animals show exacerbated dextran sodium sulphate (DSS) colitis, skewing towards pathogenic Th17 responses with reduced interleukin‐10, altered microbiota and increased invasive tumourigenesis, and bone‐marrow transplantation localises the effect to the haematopoietic compartment.^63^ This is a genuinely compartment‐resolved result and one of the few immune‐competent in vivo demonstrations in the field, but it is member‐blind: the phenotype cannot be assigned to IFITM1 or to IFITM3 individually, and the mouse cluster additionally contains paralogs, including *Ifitm6*, that have no human counterpart. The finding therefore constrains the compartment axis while leaving the member axis unfixed, and should not be cited as evidence for the direction of any single human IFITM.

Simultaneous paralog deletion demonstrates a combined requirement, not equivalence. In SiHa cervical carcinoma cells, in which IFITM2 is not expressed, deletion of IFITM1 and IFITM3 together lowers IFN‐γ‐induced HLA‐B protein and the 80S ribosomal fraction without a corresponding change in transcript level, indicating a translation‐level requirement.^64^ Two features of that experimental design bound its interpretation: the deletion removes both paralogs simultaneously, and the detection antibody recognises an epitope shared by IFITM1 and IFITM3 and cannot resolve them. The result therefore establishes that the two proteins are *jointly* required in this setting, which is consistent with the documented hetero‐oligomerisation of immunity‐related IFITMs, and is explicitly not evidence that they are functionally interchangeable. Single‐member deletion in the same system, which selectively affects some IFN‐γ‐induced outputs and not others,^45^ argues in the opposite direction. Because CRISPR guide design within a cluster of paralogs sharing more than 90% identity carries a real risk of unintended deletion at neighbouring loci, we regard sequencing confirmation of the remaining paralogs, and rescue with the individual member, as the minimum standard for member attribution in this family.

Compartment‐conditional knockout is the field's principal gap. Only one study approaches the standard: perturbation of IFITM3 restricted to FOXP3^+^ regulatory T cells, which showed that IFITM3 loss enhances STAT1 phosphorylation, drives a Th1‐like, IFN‐γ‐secreting regulatory T‐cell phenotype and licenses anti‐tumour immunity across multiple syngeneic models.^65^ No comparable cell‐type‐restricted allele has been applied to IFITM1 or IFITM2 in a tumour setting, and no study has tested a single IFITM member in an epithelial‐versus‐haematopoietic conditional design at the same anatomical site. This is precisely why the colitis‐associated CRC conflict cannot currently be resolved: the host‐protective observation comes from whole‐locus deletion in the haematopoietic compartment^63^ and the tumour‐promoting observation from epithelial tumour‐cell studies, so member and compartment are confounded by design rather than by biology. The discriminating experiments proposed in Section 9 are specified as compartment‐conditional, single‐member deletions for this reason, and the absence of such alleles is flagged as a priority evidence gap in Section 10.3.

## 3 IFITM1 AS A TUMOUR‐INTRINSIC SURFACE/SIGNALLING REGULATOR

### 3.1 Core signalling programs: Proliferation, MAPK/STAT/NF‐κB, EMT/invasion/metastasis

*Coordinates for this section*. Member: IFITM1 · Compartment: transformed tumour cell (tumour‐intrinsic) · IFN‐input: predominantly not IFN‐defined (IFITM1 studied as a MAPK/STAT/NF‐κB and EMT node; type I IFN involvement only where specified) · Tumour‐milieu: multiple epithelial lineages (colorectal, breast, lung, head‐and‐neck, laryngeal, pancreatic, ovarian, gastric) · Tier ceiling: B (see Table 1). Across colorectal, breast, lung, head‐and‐neck, laryngeal, pancreatic, ovarian and gastric carcinomas, IFITM1 has been described as a tumour‐intrinsic plasma‐membrane and surface–signalling regulator whose elevated expression supports proliferation, EMT, invasion and metastasis. The striking feature of this literature is not the breadth of tumour types involved but the limited vocabulary of partner pathways into which IFITM1 is recurrently co‐opted, and that pattern, rather than any single mechanism, anchors the interpretation that follows. Table 1 provides a systematic summary of the evidence base for IFITM1 across major solid‐tumour types.

In CRC, high IFITM1 expression correlates with poor prognosis, advanced clinical stage, and migratory, invasive, and EMT‐like phenotypes, with caveolin‐1 (CAV‐1) implicated as a downstream effector of IFITM1‐driven aggressiveness and an inverse correlate of IFITM1 in patient cohorts.^16^, ^17^ Organoid work further links IFITM1 to differential extracellular‐vesicle uptake in APC‐mutant CRC, suggesting that IFITM1 influences not only cytoskeletal and adhesion programs but also intercellular trafficking.^66^ Breast cancer evidence is more heterogeneous and exposes the importance of context: IFITM1 cooperates with the collagen receptor DDR2 to support proliferation, invasion and xenograft growth^24^; its anti‐proliferative response to inhibition in estrogen receptor (ER)‐positive cells appears p53‐dependent and is consistent with a JAK/STAT‐coupled regulatory loop^67^; in SUM149 inflammatory breast cancer cells, IFITM1‐driven aggression depends on signal transducer and activator of transcription 2 (STAT2) and the chromatin remodeller Brahma‐related gene 1 (BRG1; also known as SMARCA4)^68^; and in TNBC, interferon‐α (IFN‐α)/NF‐κB crosstalk sustains IFITM1 expression that supports tumour growth in vivo.^48^ Lung cancer studies converge on a stemness‐and‐progression axis: in non–small‐cell lung cancer (NSCLC), IFITM1‐mediated EGFR/SRY‐box transcription factor 2 (EGFR/SOX2) signalling is required for tumour formation in xenograft models,^69^ whereas in small‐cell lung cancer (SCLC) IFITM1 supports distant metastasis in orthotopic xenografts.^70^ In head‐and‐neck squamous cell carcinoma, IFITM1 marks the invasive front and is associated with upregulation of matrix‐remodelling genes^71^; in laryngeal squamous cell carcinoma (LSCC), it supports proliferation, migration and invasion via osteopontin and NF‐κB.^72^ Pancreatic cancer cell‐line work shows that IFITM1 depletion reduces proliferation, arrests the cell cycle at G1/S, induces apoptosis, and suppresses MAPK signalling.^23^ Ovarian and gastric studies add a regulatory layer: in metastatic ovarian xenografts IFITM1 is de‐repressed through promoter hypomethylation,^73^ and in gastric cancer methylation‐dependent IFITM1 expression correlates with intestinal‐type adenocarcinoma and differentiation status, indicating that IFITM1 output is itself epigenetically tunable.^74^

**FIGURE 3:**
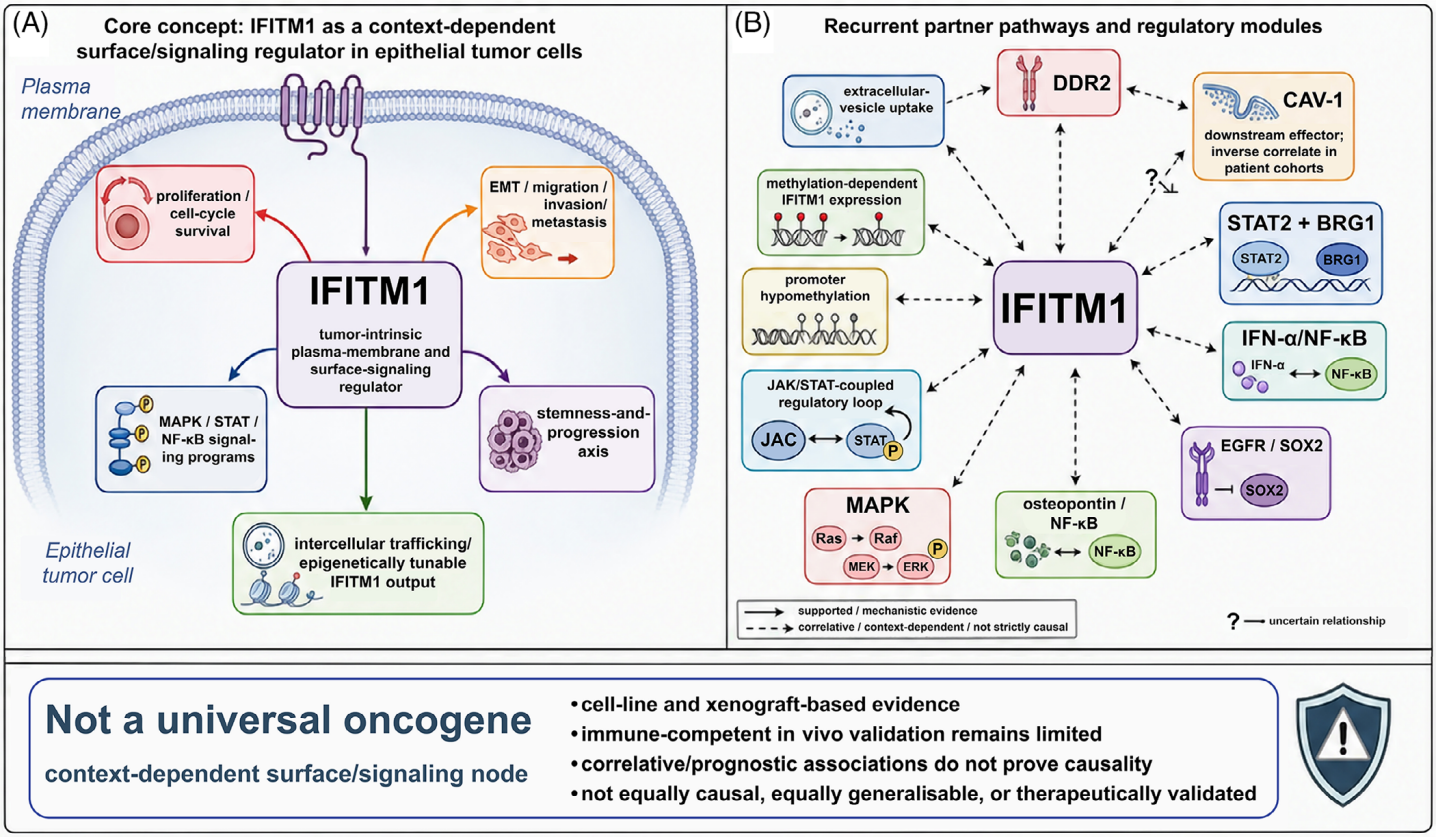
Context‐dependent tumour‐intrinsic signalling functions of interferon‐induced transmembrane 1 (IFITM1) in epithelial cancers. IFITM1 is depicted with its established topology: a single intramembrane domain and a single transmembrane domain flanking the conserved intracellular loop, with the N‐terminus cytosolic and the C‐terminus extracellular. It is not a multipass membrane protein. (A) IFITM1 acts as a plasma‐membrane and surface–signalling regulator linked to proliferation, survival, MAPK/STAT/NF‐κB signalling, epithelial–mesenchymal transition (EMT), invasion, metastasis, stemness and intercellular trafficking. (B) Recurrent partner modules include DDR2, CAV‐1, STAT2/BRG1, IFN‐α/NF‐κB, EGFR/SOX2, osteopontin/NF‐κB, MAPK, extracellular‐vesicle uptake and epigenetic regulation. The figure highlights the principal recurring mechanisms, whereas additional cancer‐specific findings and evidence limitations are detailed in Section 3.1 and Table 1.

These findings are largely cell‐line and xenograft‐based, and immune‐competent in vivo validation remains limited; correlative or prognostic associations should not be conflated with causal demonstrations, and several cancer‐type‐specific claims rest on small patient cohorts. Taken together, the evidence is best read not as proof that IFITM1 is a universal oncogene but as support for the view that it functions as a context‐dependent surface/signalling node whose recurring partners, CAV‐1, DDR2, STAT2/BRG1, IFN‐α/NF‐κB, EGFR/SOX2, osteopontin/NF‐κB and MAPK, explain its repeated association with epithelial tumour progression without implying that all IFITM1 findings are equally causal, equally generalisable or therapeutically validated (Figure 3). Read through the framework, this literature holds the member axis constant (IFITM1) and the compartment axis constant (transformed tumour cell), leaves the IFN‐input axis unmanipulated in nearly all studies, and varies only the tumour‐milieu axis across eight epithelial lineages; the framework‐level observation is therefore the recurrence of a limited partner set across a widely varied milieu, rather than any single mechanism. The tier ceiling here is B, resting on xenograft and knockdown models, and no Tier A study of tumour‐intrinsic IFITM1 currently exists.

### 3.2 Cancer‐type‐specific contexts and therapy resistance

*Coordinates for this section*. Member: IFITM1 (bidirectional: oncogenic and, in cervical squamous cell carcinoma and neurofibromatosis type 1‐associated malignant peripheral nerve sheath tumour (NF1‐MPNST), tumour‐suppressive) · Compartment: transformed tumour cell, with stromal (CAF‐exosome) and therapy‐selection inputs · IFN‐input: context‐dependent, type I IFN (endocrine/aromatase‐inhibitor resistance), radiation‐induced type I IFN (oral) and IFN‐γ (NF1‐MPNST); mixed · Tumour‐milieu: lineage‐ and therapy‐resistant settings (endocrine‐resistant breast, radioresistant oral, cisplatin‐resistant SCLC, cervical, NF1‐MPNST, bladder, anal) · Tier ceiling: B (see Table 1). The convergent partner network described in Section 3.1 neither erases cancer‐type‐specific deviation nor implies uniform IFITM1 behaviour under therapeutic pressure. IFITM1 acts as a context‐dependent surface/signalling and IFN‐response node whose direction of effect is shaped by tumour lineage, inflammatory state, stromal input and treatment selection.

Two settings deviate from a uniformly oncogenic IFITM1 model. In cervical squamous cell carcinoma, IFITM1 is downregulated relative to adjacent and normal cervical epithelium, and its restoration restrains proliferation in a manner consistent with a retained antiviral/growth‐inhibitory program.^75^ In NF1‐associated MPNSTs, IFITM1 is likewise downregulated relative to benign plexiform neurofibromas, and its restoration, or IFN‐γ exposure, reduces Ras–extracellular signal‐regulated kinase (ERK) activation in cell‐line and xenograft models.^76^ Both read as context‐specific exceptions rather than as evidence of broad tumour restraint, and both carry a control‐tissue caveat examined in Section 9. Separately, and in the oncogenic direction, surfaceome proteomics in cervical cancer cells indicates that IFITM1 reshapes membrane proteins involved in endocytosis and lysosomal transport, cell–cell and cell–matrix adhesion, antigen presentation and immune interaction, with effects on migration, invasion and immune engagement^77^; the directional opposition between^77^ and^75^ within the same lineage is analysed in Section 9.

Therapy‐resistance studies place IFITM1 within IFN‐α‐driven adaptive programs. In SCLC, elevated IFITM1 marks cisplatin‐resistant tumours and cell lines, and silencing restores chemosensitivity in vivo through Wnt/β‐catenin.^78^ In endocrine‐resistant breast cancer, PITX2 transcriptionally activates IFITM1 to potentiate IFN‐α‐induced AKT signalling and letrozole resistance,^79^ while in aromatase inhibitor (AI)–resistant cells IFITM1 suppression reduces proliferation and invasion in vivo via JAK/STAT‐mediated p21 induction.^49^ Constitutive IFITM1 and other IFN‐stimulated genes sustain this resistant phenotype, and IFITM1 knockdown sensitises cells to estrogen‐induced death.^80^

A second axis emerges around radiation and inflammatory states. In oral cancer, combining IFITM1 knockdown with radiotherapy suppresses xenograft growth and remodels STAT1/2/3–p21 signalling.^81^ In oral squamous cell carcinoma (OSCC), carcinoma‐associated fibroblast (CAF)‐derived exosomal long non‐coding RNA RORA‐AS1 binds IFITM1 transcripts and drives radio‐resistance via an IFITM1/STAT axis.^82^ In anal squamous cell carcinoma (ASCC), IFITM1 belongs to a broader IFN‐α/IFN‐γ, tumour necrosis factor α/NF‐κB, EMT and inflammatory‐response program enriched in chemoradiotherapy non‐responders.^83^ In bladder cancer cell lines, constitutive type I IFN activation marks resistance to IFN‐α‐induced cytotoxicity, a state‐level concept, not direct IFITM1 causality.^84^

**FIGURE 4:**
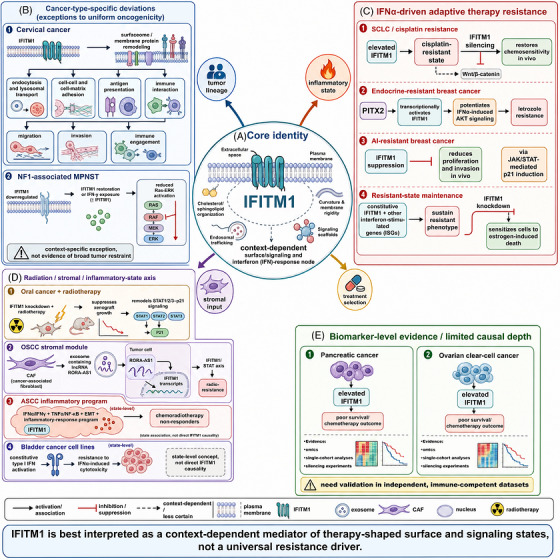
Cancer‐type‐specific and therapy‐shaped functions of interferon‐induced transmembrane 1 (IFITM1). (A) IFITM1 acts as a context‐dependent membrane, signalling and interferon (IFN)‐response node. (B) Cervical cancer and neurofibromatosis type 1‐associated malignant peripheral nerve sheath tumour (NF1‐MPNST) illustrate lineage‐specific deviations from uniform oncogenic activity. (C, D) IFITM1 contributes to cisplatin, endocrine, aromatase‐inhibitor and radiotherapy resistance through Wnt/β‐catenin, AKT, JAK/STAT–p21, stromal‐exosomal and inflammatory programs. (E) Elevated IFITM1 also shows biomarker‐level associations with adverse outcomes in pancreatic and ovarian clear‐cell cancers. The figure highlights selected principal mechanisms; additional studies and context‐specific details are provided in Section 3.2 and Table 1.

Pancreatic^85^ and ovarian clear‐cell^86^ studies add biomarker‐level evidence that elevated IFITM1 associates with poor survival or chemotherapy outcome, but rest on omics, single‐cohort analyses and silencing experiments that need validation in independent, immune‐competent datasets. Across these contexts, IFITM1 is best interpreted as a context‐dependent mediator of therapy‐shaped surface and signalling states, not as a universal resistance driver (Figure 4). Within the framework, the member and compartment axes remain fixed (IFITM1; transformed tumour cell), and the decisive variable is prior‐treatment exposure within the tumour‐milieu axis, with the IFN‐input axis only partially specified: type I IFN in the endocrine‐ and aromatase‐inhibitor‐resistant settings, radiation‐induced type I IFN in oral cancer, and IFN‐γ in NF1‐MPNST. The tier ceiling is again B, and the two directionally opposite settings (cervical carcinoma and NF1‐MPNST) vary the milieu axis rather than the member or compartment axis, which is why they are read as lineage‐specific deviations and not as evidence of family‐level ambiguity.

## 4 IFITM3 FROM HCC TO HAEMATOLOGIC MALIGNANCIES

### 4.1 HCC and gastrointestinal paradigm

*Coordinates for this section*. Member: IFITM3 · Compartment: transformed tumour cell (including germline‐variant carriers) · IFN‐input: predominantly not IFN‐defined (germline rs12252 variants, non‐coding‐RNA control, ERK1/2–c‐MYC, hepatocyte growth factor receptor (MET)/AKT, Wnt); chronic viral (HCV/hepatitis B virus (HBV)) disease context for the single‐nucleotide polymorphism (SNP) studies · Tumour‐milieu: hepatocellular carcinoma (HCC) and gastrointestinal epithelia (gastric, colon) · Tier ceiling: B, gastric and colon functional models reach Tier B, whereas the HCC literature is predominantly Tier C–D with two Tier B functional exceptions (see Table 2). IFITM3 biology in HCC and gastrointestinal cancers reflects a layered model in which IFITM3 abundance, germline variation, non‐coding RNA regulation, and kinase or transcriptional outputs shape progression. These converge on extracellular signal‐regulated kinase 1/2 (ERK1/2)–c‐MYC, phosphoinositide 3‐kinase/protein kinase B/mammalian target of rapamycin (PI3K/AKT/mTOR), MET/AKT/forkhead box O3 (FOXO3)/c‐MYC, Wnt/β‐catenin and EMT programs, with Krüppel‐like factor 4 (KLF4) acting upstream in colon tissue. Table 2 provides a systematic summary of the evidence base for IFITM3 across HCC, gastrointestinal cancers and other solid tumours to haematologic malignancies.

The HCC literature is dominated by association‐level evidence. Cohort studies report IFITM3 overexpression in HCC tissue^87^ and link IFITM3 polymorphisms to risk, including the IFITM3/matrix metalloproteinase‐9 (MMP‐9) co‐polymorphism^88^ and the rs12252‐CC genotype enriched in HCC patients and associated with lower differentiation, larger tumours and higher relapse.^89^ IFNL4–IFITM3 variants suggest a risk‐linked context in HCV‐related cirrhosis and HCC,^90^ while IFITM3 rises across HCC progression and correlates with rs12252‐CC and a PI3K/AKT/mTOR signature.^91^ These associations support risk stratification but do not establish causality.

Mechanistic HCC studies converge on non‐coding RNA control of IFITM3 and on ERK1/2–c‐MYC output. miR‐29a represses IFITM3 and constrains migration, invasion, proliferation and survival in cell‐line and xenograft models,^92^ with miR‐142‐5p exerting a similar restraint.^93^ Two long non‐coding RNA (lncRNA) lift this restraint: TUG1 acts as a competing endogenous RNA for miR‐29a, derepressing IFITM3,^94^ and CKMT2‐AS1 sponges miR‐142‐5p to the same effect.^93^ Downstream, IFITM3 sustains c‐MYC via ERK1/2 to promote HCC proliferation.^95^ These pathways are mechanistically supported, but cross‐study replication and independent‐cohort validation remain limited.

Gastric cancer offers the strongest mechanistic anchor. Quantitative proteomics positions IFITM3 as a MET–AKT scaffold that suppresses FOXO3 and induces c‐MYC, driving malignant progression, stemness, metastasis and chemoresistance in vivo.^28^ A complementary study links IFITM3 overexpression to proliferation, migration, invasion, cell‐cycle progression, Wnt/β‐catenin activity, EMT reversal on silencing, and reduced MMP‐2/MMP‐9.^96^ These provide a deeper multi‐pathway link than most HCC association studies, though both rest on cell‐line and short‐cohort evidence.

In colon cancer, IFITM3 is mechanistically tethered to KLF4. KLF4 binds the IFITM3 promoter and represses transcription; KLF4 loss in human tumours and Villin‐Cre Klf4‐null mice yields IFITM3 overexpression, and knockdown reduces proliferation, migration, invasion and xenograft growth.^15^ Chinese‐cohort profiling finds IFITM3 elevated in adenoma, supporting relevance across the adenoma–carcinoma trajectory,^97^ biomarker‐level support, not proof of causal necessity.

An advanced‐HCC conference abstract presented at American Association for Cancer Research (AACR) 2025 nominates exosomal IFITM3 as a candidate stratification marker^98^ (abstract‐level evidence, not peer‐reviewed). Read through the framework, these studies hold the member axis constant (IFITM3) in the tumour‐cell compartment and vary the tumour‐milieu axis: the same protein drives ERK1/2–c‐MYC in HCC, a MET–AKT–FOXO3 scaffold in gastric cancer, and a KLF4‐repressed program in colon, with the IFN‐quality axis left unmanipulated in all of them. The evidence is therefore milieu‐resolved but IFN‐quality‐blind, and is strongest where in vivo perturbation exists (gastric and colon models, Tier B) rather than in the association‐dominated HCC literature (Tier C–D).

### 4.2 Other solid tumours, haematologic malignancies and targeted‐agent resistance

*Coordinates for this section*. Member: IFITM3 · Compartment: transformed tumour cell (with glioblastoma stem cell [GSCs]–endothelial crosstalk in glioblastoma multiforme (GBM); leukaemic cell in AML/chronic myeloid leukaemia (CML)) · IFN‐input: predominantly not IFN‐defined (kinase‐ and membrane‐associated rewiring); therapy‐resistance context in clear‐cell renal cell carcinoma (ccRCC) and EGFR‐mutant NSCLC · Tumour‐milieu: GBM, prostate, OSCC, breast, ccRCC, EGFR‐mutant NSCLC, AML, CML · Tier ceiling: B, stronger support in solid tumours (ccRCC, EGFR‐NSCLC) than in the correlative haematologic analyses (see Table 2). Beyond 4.1, IFITM3 evidence reaches GBM, prostate cancer, OSCC, breast cancer, ccRCC, epidermal growth factor receptor (EGFR)‐mutant NSCLC, AML and CML. Across these settings, IFITM3 acts as a context‐dependent kinase‐ and membrane‐associated rewiring node, with stronger support in solid tumours than in haematologic correlative analyses.

In GBM, IFITM3 in GSCs activates JAK/STAT3 signalling and drives basic fibroblast growth factor (bFGF) secretion, supporting tumour angiogenesis.^99^ In prostate cancer, IFITM3 binds SMAD4 to engage transforming growth factor‐β (TGF‐β)–SMAD signalling and a MAPK arm, promoting EMT and bone‐tropic metastasis.^18^ In OSCC, IFITM3 knockdown lowers CCND1 and CDK4, reduces RB phosphorylation, and induces senescence and apoptosis.^100^ In non‐TNBC, IFITM3 knockdown impairs cell growth, colony formation and G0/G1‐to‐S progression.^101^ These outputs are tumour‐type‐specific, not a universal pathway.

Targeted‐agent resistance is the strongest conceptual bridge. In ccRCC, IFITM3 is elevated in post‐tyrosine kinase inhibitor (TKI) samples, and sunitinib‐resistant cells display proliferative, clonogenic, migratory and invasive features that IFITM3 knockdown suppresses in vitro and in xenografts via tumour necrosis factor receptor‐associated factor 6 (TRAF6)/MAPK/activator protein 1 (AP‐1).^47^ In EGFR‐mutant NSCLC, IFITM3 is upregulated in poor osimertinib responders and rises further during treatment in response to TME /cytokines; IFITM3 interacts with MET to activate AKT, and MET inhibition suppresses resistance in xenografts.^60^ Both place IFITM3 at a kinase‐rewiring interface through distinct partners, TRAF6/MAPK/AP‐1 versus MET/AKT.

Haematologic evidence is mixed. High IFITM3 predicts shorter event‐free and overall survival in chemotherapy‐treated AML, prognostic, not mechanistic, and the effect disappears after allogeneic transplantation.^22^ A preclinical study combining iron oxide nanoparticles IONPs with cytarabine (Ara‐C) reduces IFITM3, proliferation, clonogenicity, AML markers and disease burden in mice^102^; preclinical, not clinically validated. In CML, exosomes from imatinib‐resistant K562 cells carry IFITM3, CD146 and CD36 and increase survival of sensitive cells under imatinib, an exosomal‐transfer phenomenon distinct from AML.^103^ A multi‐omics endoplasmic reticulum stress AML signature^104^ is sometimes cited here but contains IFITM1, not IFITM3, a cautionary example against family‐level misattribution.

Within the framework, these reports again fix the member (IFITM3) and compartment (tumour cell) and vary the milieu, with prior‐treatment exposure emerging as the decisive milieu variable: IFITM3 marks the post‐TKI and post‐osimertinib resistant state through distinct partners—TRAF6/MAPK/AP‐1 in ccRCC and MET/AKT in NSCLC (both Tier B). Haematologic evidence sits lower on the tier scale (prognostic AML association, Tier D; exosomal transfer in CML, Tier C) and, as the ER‐stress signature that in fact carries IFITM1 rather than IFITM3 shows, is where member misattribution is most likely.

## 5 IFITM2 AND THE LESS‐CHARACTERISED MEMBERS (IFITM5, IFITM10)

### 5.1 IFITM2

*Coordinates for this section*. Member: IFITM2 · Compartment: transformed tumour cell, with lymphatic‐endothelial and NK‐effector crosstalk · IFN‐input: predominantly not IFN‐defined (PI3K/AKT, TGF‐β/SMAD, IGF1/STAT3, NF‐κB); one IFN‐γ‐defined context (CD8‐derived IFN‐γ in elderly GBM) · Tumour‐milieu: colorectal, gastric, ccRCC, PDAC, elderly glioblastoma · Tier ceiling: B (gastric in vivo and CRISPR studies; see Table 3). IFITM2 should not be folded into family‐level IFITM claims. Its cancer literature is smaller and less mature than IFITM1's or IFITM3's, but recurring links to proliferation, invasion, EMT and lymph‐node and distant metastasis, and inflammatory or growth‐factor signalling justify a member‐specific narrative. Table 3 provides a systematic summary of the evidence base for IFITM2 and the less‐characterised members IFITM5 and IFITM10.

In CRC, IFITM2 is overexpressed, correlates with poor prognosis, and supports proliferation, migration and invasion through phosphoinositide 3‐kinase/protein kinase B (PI3K/AKT) activation.^105^ Gastric cancer (GC) studies extend this picture along three convergent axes: IFITM2 supports TGF‐β1‐driven EMT via SMAD family members 2 and 3 (SMAD2/3)^25^; insulin‐like growth factor 1 (IGF1)/IGF1 receptor (IGF1R)/signal transducer and activator of transcription 3 (STAT3) signalling induces IFITM2, which sustains a feed‐forward interleukin‐6 loop and promotes growth, recurrence, and lung metastasis in vivo^106^; and integrative single‐cell and methylation profiling links IFITM2 upregulation in early gastric epithelial cells, reduced promoter methylation, and CRISPR/siRNA dependency to NF‐κB activation.^62^ Together GC and CRC offer the clearest tumour‐cell IFITM2 narrative, without a single shared pathway.

In ccRCC, elevated IFITM2 is associated with shorter survival and lymphatic metastasis, and co‐culture work points to a lymphangiogenic effect on endothelial cells; the evidence is largely prognostic and cell‐line based, not causal.^107^ In pancreatic ductal adenocarcinoma (PDAC), tumour‐secreted BCL2‐associated athanogene‐3 (BAG3) engages IFITM2 to activate MAPK/phospho‐ ERK and promote growth; a preliminary observation that secreted BAG3 dampens NK cell cytotoxicity should not be overextended into a developed immune mechanism.^108^ In elderly glioblastoma (GBM), interferon‐γ (IFN‐γ) released within an inflammatory TME triggers IFITM2 expression and a malignant phenotype, a finding tied to age and TME state, not GBM in general.^109^ Outside cancer, IFITM2 supports osteogenic differentiation of C3H10T1/2 mesenchymal stem cells via Wnt‐related programs,^110^ reminding that IFITM2 expression in mesenchymal‐rich TMEs may reflect lineage biology rather than tumour‐cell activity, a confounder rarely acknowledged in cancer studies. IFITM2 is best interpreted as a member‐specific, context‐dependent signalling amplifier centred on PI3K/AKT, TGF‐β/SMAD, BAG3/MAPK, IGF1/STAT3 and NF‐κB‐linked programs, while its biomarker and therapeutic relevance remain less mature than that of IFITM1 and IFITM3. Read through the framework, the IFITM2 literature fixes the member axis and holds the compartment axis predominantly at the transformed tumour cell, leaves the IFN‐input axis undefined in every study except elderly glioblastoma, where CD8^+^‐T‐cell‐derived IFN‐γ is the specified input, and varies the tumour‐milieu axis across colorectal, gastric, renal, pancreatic and glial lineages. The tier ceiling is B, set by the gastric in vivo and CRISPR studies, and no immune‐competent in vivo perturbation of IFITM2 has been reported, so the compartment axis for this member remains effectively untested outside the tumour cell.

### 5.2 IFITM5 and IFITM10

*Coordinates for this section*. Member: IFITM5 and IFITM10 (non‐IFN‐inducible paralogs) · Compartment: transformed tumour cell (osteoblast‐like for IFITM5) · IFN‐input: N/A, these members are not IFN‐inducible and are not placed on the acute/chronic IFN axis · Tumour‐milieu: osteosarcoma (OS; IFITM5); colorectal and gastric (IFITM10) · Tier ceiling: C. Note directional divergence: IFITM5 is tumour‐suppressive/pro‐differentiative, and gastric IFITM10 decreases with T stage (see Table 3). IFITM5 and IFITM10 are grouped here not because they are functionally equivalent but because their cancer literature is too small to support separate, expansive sections. This consolidation preserves member‐level specificity while preventing the overinterpretation that one or two papers cannot sustain. The only direct IFITM5 cancer study is in vitro: SaOS2 OS cells transfected with wild‐type IFITM5 or the IFITM5 c.‐14C>T mutation shows increased apoptosis, decreased invasion and enhanced osteogenic differentiation, with elevated alkaline phosphatase (ALP), osteocalcin and runt‐related transcription factor 2 (Runx2) and more mineralised nodules; the c.‐14C>T variant additionally reduces migration.^111^ This is single‐study, single‐cell‐line evidence in an osteoblast‐lineage tumour and should not be extrapolated to other sarcomas or epithelial cancers.

IFITM10 evidence comprises two studies in distinct gastrointestinal contexts. In CRC, IFITM10 is upregulated, correlates with angiogenic features, and is tied to STAT3 phosphorylation, with IFITM10 silencing impairing endothelial tube formation.^112^ In GC, The Cancer Genome Atlas (TCGA) and patient‐tissue quantitative polymerase chain reaction (qPCR) analyses show high IFITM10 expression with strong receiver operating characteristic curves for distinguishing tumour from adjacent tissue, and an association with earlier T stage.^113^ The CRC report provides a mechanistic angiogenesis signal, whereas the GC report is biomarker‐level association without comparable mechanistic depth.

Both are biologically meaningful but evidence‐thin. The IFITM10–STAT3 angiogenesis link rests on a single CRC study and requires independent validation; the GC diagnostic association does not establish causality; and the IFITM5 finding is a single in vitro OS report. IFITM5 and IFITM10 should be retained as member‐specific but evidence‐limited IFITM proteins whose cancer relevance is hypothesis‐generating rather than generalisable or clinically validated. Read through the framework, IFITM5 and IFITM10 sit off Axis 2 entirely: because neither is IFN‐inducible, the acute‐versus‐chronic IFN‐input distinction that organises Sections 4.2, 5.1, 5.2 is not defined for them, which is itself a prediction of the framework rather than a gap in the evidence. The member and compartment axes are fixed (osteoblast‐like tumour cell for IFITM5; tumour cell with endothelial crosstalk for IFITM10) and only the milieu axis varies, with a tier ceiling of C for both members.

## 6 THE ACUTE–CHRONIC DICHOTOMY OF INTERFERON SIGNALLING AS THE DETERMINANT OF IFITM FUNCTIONAL OUTPUT

*Coordinates for this section*. Member: IFITM1, IFITM2 and IFITM3 (member‐resolved instantiations) · Compartment: transformed tumour cell, with immune‐compartment cross‐references · IFN‐input: this section defines the axis itself (acute/transient vs. chronic/sustained) · Tumour‐milieu: cross‐lineage (SCLC, TNBC, gastric, ccRCC, EGFR‐mutant NSCLC) · Tier ceiling: A (SCLC IFITM3–MHC‐I and TNBC IFITM3–PD‐L1 anchors; see Tables 4 and 5). We use ‘acute’ and ‘chronic’ IFN input in an operational sense. Acute/transient signalling denotes a single or short (hours to ∼72 h) exposure to exogenous IFN‐γ or type I IFN, read out as STAT1/ interferon regulatory factor 1 (IRF1)‐dependent induction of MHC‐I, β2‐microglobulin and antigen‐processing components. Chronic/sustained signalling denotes sustained or repeated exposure over days to weeks, or constitutive tumour‐cell‐intrinsic ISG expression driven by stromal/CAF‐ or microbiota‐derived IFN, read out as persistent STAT1/IRF1 activity, PD‐L1 upregulation, SOCS1/3 and PTPN2 induction, and the ISG.RS/interferon‐related DNA‐damage‐resistance signature (IRDS). Neither pole should be confused with tonic IFN signalling, which denotes the constitutive, low‐amplitude type I IFN tone of healthy steady‐state tissue (Section 2.3) and functions as the baseline rather than as a tumour‐associated regime; the studies discussed below are classified as acute or chronic by their experimental IFN input and downstream readout, not by their baseline ISG tone. Crucially, the level of an IFITM protein or of ISGs, taken alone, does not indicate which regime is active: the discriminating readout is the downstream program, MHC‐I/antigen‐presentation gain (sensitisation) versus PD‐L1/ISG.RS engagement (escape), not IFITM abundance. Throughout Sections 5.1, 5.2, 8 we therefore assign direction of effect from these downstream readouts rather than from IFITM expression. IFN‐γ is a pleiotropic cytokine whose role in the TME is fundamentally dual, supporting tumour regression under some conditions and treatment resistance under others.^114^ In its canonical and best‐established form, however, IFN signalling, both type I (IFN‐α/β) and type II (IFN‐γ), is a positive regulator of antigen presentation and tumour immunogenicity.^115^, ^116^ Mechanistically, IFN‐γ engages the JAK1/2–STAT1 cascade to induce IRF1, which transactivates MHC‐I genes and thereby increases the susceptibility of tumour cells to cytotoxic T‐cell recognition.^115^ The same cytokine upregulates the class II transactivator (CIITA) to drive MHC class II expression, enhances cytotoxic T‐cell and NK‐cell activity, and promotes dendritic‐cell maturation, collectively reinforcing adaptive priming.^114^, ^115^ Critically for the present context, in TNBC the immunologically ‘cold’, lymphocyte‐poor phenotype arises not from IFN signalling itself but from its suppression: MYC, together with its co‐repressor MIZ1, directly binds and downregulates IFN‐pathway genes, and restoring this signalling reverses the immune‐excluded state and sensitises tumours to STING‐driven immunity.^117^ The IFN response is therefore most accurately characterised as a master determinant of tumour immunogenicity whose default direction is pro‐immunogenic.^115^, ^117^

This pro‐immunogenic role, however, is conditional on the temporal quality and amplitude of the signal, and it is this distinction, rather than the presence or absence of IFN, that reconciles the apparently opposing reports in the IFITM literature. Acute, transient IFN‐γ exposure drives the STAT1/IRF1‐dependent antigen‐presentation and effector‐recruitment program described above, whereas prolonged exposure becomes maladaptive, upregulating immune‐checkpoint molecules such as PD‐L1, triggering T‐cell exhaustion and recruiting regulatory T cells in a manner linked to therapeutic resistance.^114^, ^118^ Under such chronic stimulation, constitutive STAT1/IRF1 activity additionally sustains negative regulators including SOCS1/3 and PTPN2, entrenches an epigenetically stabilised exhaustion program, and can culminate in antigen‐presentation loss through β2‐microglobulin/HLA‐I or JAK1/2 defects, yielding tumours that are ‘inflamed but ineffective’.^118^ This sustained‐ signalling state corresponds to the IFN‐stimulated gene resistance signature (ISG.RS) that confers multigenic resistance to checkpoint blockade.^119^ Input magnitude operates in parallel: low‐dose IFN‐γ can paradoxically promote tumour‐cell stemness and metastatic behaviour, whereas high‐dose exposure favours regression, so that local IFN‐γ concentration itself helps set the sign of the effect.^115^ Prolonged or elevated signalling also exerts selective immune pressure that downregulates MHC‐I and other antigen‐processing components, the core of the ‘interferon‐gamma paradox’ in cancer.^120^ The same biphasic principle extends beyond oncology: in human viral infection, sustained IFN signatures track with impaired antigen presentation and delayed adaptive immunity, exemplifying an ‘interferon paradox’ in which timing, not quantity, dictates outcome.^121^ Applied to the IFITM literature, this acute–chronic axis is not merely inferential; it is instantiated by member‐resolved data developed in the following sections. At the acute, immunogenic pole, IFITM3 supports NLR family CARD‐domain‐containing protein 5 (NLRC5)‐driven MHC‐I induction, CD8^+^ T‐cell infiltration and anti‐programmed cell death protein 1 (PD‐1) responsiveness in SCLC (Section 7).^50^ At the chronic pole, sustained NF‐κB‐coupled signalling redirects IFITMs towards checkpoint engagement, histone H3 lysine 27 acetylation (H3K27ac)–IFITM3–TNF‐α/NF‐κB/PD‐L1 in TNBC^61^ and IFITM2–JAK/STAT3/PD‐L1 in gastric cancer^122^ (Section 8.3), and the same chronic arm is evident where IFITM3 rises in tyrosine‐kinase‐inhibitor–resistant ccRCC^47^ and osimertinib‐resistant EGFR‐mutant lung cancer^60^ (Section 4.2). The direction of the IFITM effect therefore tracks the temporal quality of the IFN input rather than IFITM abundance itself.

Accordingly, we frame IFITM behaviour around this acute‐versus‐chronic axis throughout the remainder of this manuscript, and the TNBC findings discussed below are interpreted as a manifestation of the chronic, PD‐L1‐coupled arm of IFN signalling rather than as evidence that IFN signalling is intrinsically immunosuppressive.^117^, ^118^

### 6.1 The IRDS: A mechanistic bridge between chronic IFN signalling and IFITM‐associated resistance

*Coordinates for this section*. Member: IFITM1, IFITM2 and IFITM3 (as IFN‐stimulated constituents; not individually resolved) · Compartment: transformed tumour cell · IFN‐input: chronic, low‐dose type I IFN (U‐STAT1–IRF9‐driven) · Tumour‐milieu: cross‐lineage · Tier ceiling: not applicable; the IRDS literature is not IFITM‐resolved and is cited as upstream mechanistic context. The IRDS provides a defined mechanistic bridge connecting the chronic arm of this axis to IFITM biology. The IRDS is a set of constitutively expressed ISGs whose sustained expression, driven by chronic, low‐dose type I IFN acting through unphosphorylated STAT1 and an IRF9‐dependent program rather than by transient STAT1 phosphorylation, confers resistance to DNA‐damaging chemotherapy and radiation across multiple cancer types.^123^, ^124^, ^125^ Because the IRDS and the checkpoint‐blockade‐resistance signature (ISG.RS) share this chronic‐IFN origin and were defined by the same investigators,^119^ therapy resistance and adaptive immune resistance can be read as two outputs of one sustained‐signalling state. IFITMs are themselves ISGs, and their persistent expression under chronic IFN input is most parsimoniously interpreted as a component and readout of this IRDS‐type program. This framing reconciles three otherwise disconnected strands of the IFITM cancer literature: the elevation of IFITM3 in targeted‐therapy‐resistant tumours (Section 4.2), its coupling to PD‐L1 under chronic NF‐κB‐driven signalling (Section 8.3), and the acute‐versus‐chronic reversal of its immunological effect (Sections 7 and 8.3). We therefore propose that chronic, IRDS‐type IFN signalling is the upstream determinant unifying the therapy‐resistance and immune‐resistance faces of IFITM biology, and we flag direct, IFITM‐resolved testing of this hypothesis, specifically, whether individual IFITM members are functional IRDS constituents or co‐regulated markers, as a priority gap (Section 10.3).

## 7 IFITMS IN ANTIGEN PRESENTATION, MHC REGULATION AND INNATE IMMUNE RECOGNITION

*Coordinates for this section*. Member: IFITM3 (SCLC) and IFITM1, with two combined IFITM1/IFITM3 deletion studies · Compartment: transformed tumour cell, with CD8^+^ T‐cell and NK‐cell crosstalk · IFN‐input: acute/transient IFN‐γ where experimentally defined^45^, ^64^; not IFN‐defined in the remaining studies, in which the acute pole is inferred from the downstream antigen‐presentation readout rather than from a manipulated IFN exposure · Tumour‐milieu: SCLC, TNBC, cervical carcinoma, bladder and pan‐cancer, head‐and‐neck · Tier ceiling: A (SCLC IFITM3–MHC‐I; see Table 4). Antigen presentation by MHC‐I molecules, designated HLA‐I in humans, supports CD8^+^ T‐cell recognition, whereas MHC‐I abundance also shapes NK‐cell surveillance. The literature collected here positions IFITM1 and IFITM3 as conditional regulators of these immune‐recognition interfaces, with evidence strength differing sharply by IFITM member, tumour context and regulatory layer.

The strongest evidence concerns IFITM3 in SCLC. A set of closely related reports, treated here as a single evidence unit, shows that IFITM3 expression correlates with MHC‐I expression in SCLC cohorts; IFITM3 overexpression enriches antigen‐presentation pathways via NLRC5, the MHC class I transactivator, increases CD8^+^ T‐cell infiltration and cytotoxicity, and prolongs progression‐free survival under chemoimmunotherapy but not chemotherapy alone; immunohistochemical H‐scoring stratifies clinical outcomes, and pharmacological IFITM3 induction sensitises mouse SCLC models to anti‐PD‐1.^50^ A complementary pan‐cancer analysis links IFITM3 to inflamed TME signatures, predicting higher response to immunotherapy across several lineages, a correlative biomarker rather than a validated decision tool.^126^

A distinct mechanism operates in TNBC. In TNBC cell lines, IFITM1 overexpression is coupled to Wnt/β‐catenin activity and to decreased HLA class I, with the lowered HLA‐I sensitising tumour cells to NK‐mediated lysis in a pattern consistent with missing‐self recognition; β‐catenin or JAK inhibition restores HLA‐I and attenuates this effect.^27^ Importantly, this IFITM1‐driven, Wnt/β‐catenin‐coupled reduction of HLA‐I is a tumour‐cell‐intrinsic mechanism distinct from, and not in contradiction with, the canonical IFN‐γ‐driven induction of MHC‐I established in Section 6, illustrating that an individual ISG can modulate the antigen‐presentation machinery in a direction opposite to that of the upstream IFN signal. This is cell‐line evidence in one breast‐cancer subtype and should not be extrapolated to NK biology more broadly, to other breast‐cancer subtypes, or to IFITM3, and the lowered‐HLA‐I direction also implies the opposite consequence for CD8^+^ T‐cell recognition.

A third regulatory layer is splicing‐ and translation‐linked rather than transcription‐linked. In SiHa cervical carcinoma cells, a streptavidin‐binding peptide (SBP)‐tagged IFITM1 bait identified serine/arginine‐rich splicing factor 1 (SRSF1) as an IFITM1‐associated partner; on IFN‐γ stimulation the endogenous IFITM pool associates with SRSF1 and with HLA‐B mRNA, and simultaneous IFITM1/IFITM3 deletion lowers IFN‐γ‐induced HLA‐B protein and the 80S ribosomal fraction without a corresponding transcript‐level change, indicating a translation‐level effect on HLA‐B distinct from the IFITM3‐driven NLRC5–MHC‐I stabilisation seen in SCLC.^64^ This study does not establish that IFITM1 and IFITM3 are interchangeable: its detection antibody recognises an epitope shared by the two paralogs and cannot resolve them, and its knockout removes both members simultaneously (IFITM2 is not expressed in SiHa). It therefore demonstrates a *combined* IFITM1/IFITM3 requirement for HLA‐B translation in this context, consistent with the known hetero‐oligomerisation of immunity‐related IFITMs, rather than functional equivalence between the two.^64^ A separate head‐and‐neck cancer study identifies MORC3 as a repressor of PD‐L1 whose knockdown induces IFITM1, IFITM3 and PD‐L1; this is contextual checkpoint biology, not direct evidence of IFITM‐mediated antigen‐presentation control.^127^

**FIGURE 5:**
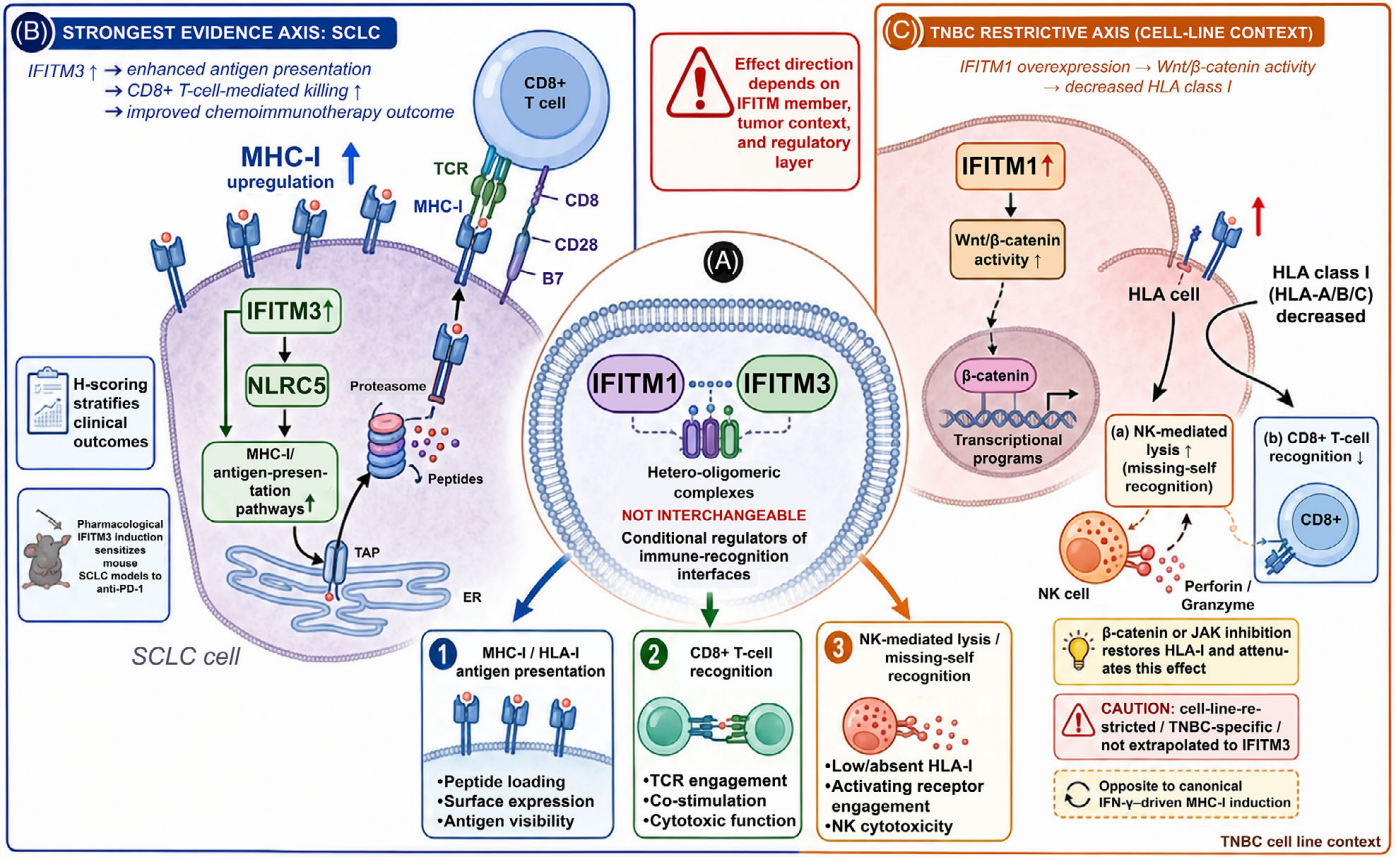
Context‐dependent regulation of antigen presentation and immune recognition by interferon‐induced transmembrane (IFITM) proteins. (A) IFITM1 and IFITM3 act as non‐interchangeable regulators of major histocompatibility complex class I (MHC‐I) antigen presentation, designated human leukocyte antigen class I (HLA‐I) in human tumours, CD8^+^ T‐cell recognition and NK‐cell surveillance. (B) In small‐cell lung cancer (SCLC), IFITM3 promotes the NLRC5–MHC‐I axis, CD8^+^ T‐cell activity and improved chemoimmunotherapy response. (C) In triple‐negative breast cancer (TNBC) cell lines, IFITM1 is associated with Wnt/β‐catenin activation and reduced HLA‐I, enhancing NK‐mediated lysis. Additional mechanisms and contextual findings are detailed in Section 7.

Across these reports the asymmetry is conspicuous: SCLC IFITM3–MHC‐I is supported by patient cohorts, mechanism and in vivo immunotherapy data; TNBC IFITM1–HLA‐I–NK is cell‐line‐restricted; cervical SRSF1–IFITM1/3–HLA‐B is mechanistically novel but tumour‐type‐restricted; and the pan‐cancer inflamed‐TME and MORC3 contributions are contextual. IFITMs can support immunogenicity through MHC/HLA‐linked and splicing‐linked mechanisms, but the strength and direction of this effect remain member‐specific, tumour‐context‐specific and dependent on evidence that is currently strongest for IFITM3–MHC‐I in SCLC (Figure 5).

## 8 IFITMS ACROSS IMMUNE COMPARTMENTS, STROMA AND ADAPTIVE IMMUNE RESISTANCE

### 8.1 Immune‐cell‐intrinsic IFITM function: Myeloid, T‐cell, B‐cell and NK compartments

**FIGURE 6:**
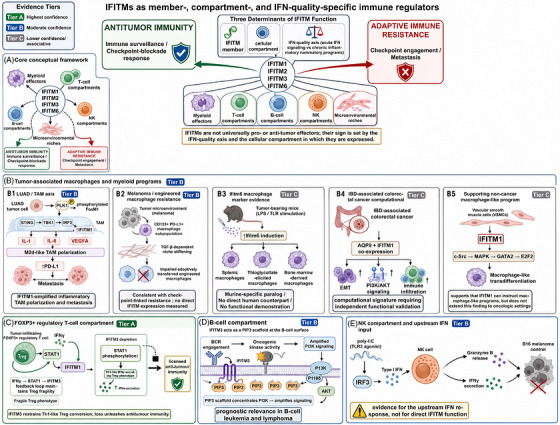
Member‐, compartment‐ and interferon (IFN)‐quality‐specific regulation of tumour immunity by interferon‐induced transmembrane (IFITM) proteins. (A) Conceptual framework showing that IFITM1, IFITM2, IFITM3 and the murine paralog Ifitm6 can exert either anti‐tumour or tumour‐promoting effects depending on the IFITM member, the cellular compartment and the quality of the upstream interferon signal. (B) Selected myeloid mechanisms include the IFITM1‐amplified STING–TBK1–IRF3 inflammatory program in lung adenocarcinoma (LUAD)‐associated macrophages, checkpoint‐linked resistance of engineered macrophages in melanoma, inducible murine Ifitm6 expression, the AQP9–IFITM1 computational signature in IBD‐associated colorectal cancer, and non‐cancer evidence for IFITM1‐driven macrophage‐like transdifferentiation. (C–E) Major lymphoid and innate axes include the IFNγ–STAT1–IFITM3 circuit controlling FOXP3^+^ Treg fragility, IFITM3‐dependent PIP3 scaffolding and PI3K amplification in B cells, and the IRF3‐dependent type I IFN response that supports natural killer (NK)‐cell effector activity and melanoma control. Evidence tiers indicate the relative confidence and directness of the available data. Because the evidence base is extensive and heterogeneous, the figure selectively presents the principal mechanistic and compartment‐specific findings, whereas additional studies, contextual associations and lower confidence observations are discussed in Section 8.1 and summarised in Table 5. Human and murine findings are presented in visually separated fields, and the mouse‐restricted paralog Ifitm6 is labelled as having no human counterpart, so that murine observations cannot be read as human mechanisms.

**FIGURE 7:**
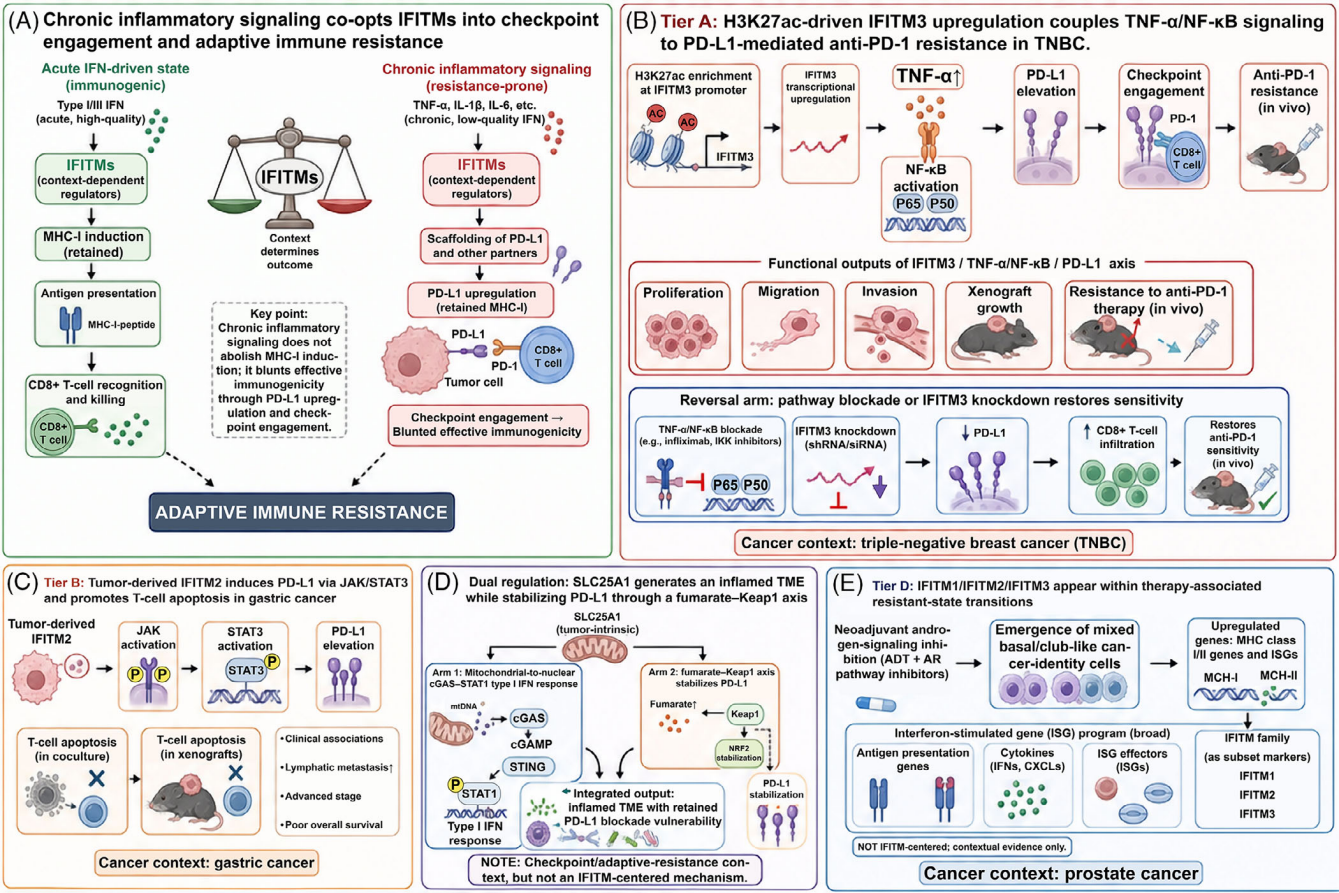
Interferon‐induced transmembranes (IFITMs) in immune‐checkpoint engagement and adaptive immune resistance. (A) In the checkpoint/adaptive‐resistance compartment, chronic inflammatory signalling co‐opts IFITMs into programmed death‐ligand 1 (PD‐L1)‐linked checkpoint engagement, contrasting with the acute IFN‐driven immunogenic state associated with major histocompatibility complex class I (MHC‐I) induction. (B) In triple‐negative breast cancer (TNBC), Tier A evidence identifies an H3K27ac–IFITM3–TNF‐α/NF‐κB–PD‐L1 axis that promotes tumour progression and anti‐PD‐1 resistance, while pathway blockade or IFITM3 knockdown restores sensitivity. (C) In gastric cancer, Tier B evidence shows that tumour‐derived IFITM2 activates JAK/STAT3, elevates PD‐L1 and promotes T‐cell apoptosis. (D, E) Additional contextual evidence includes SLC25A1‐linked inflamed PD‐L1 regulation and therapy‐associated IFITM/IFN‐stimulated gene (ISG) upregulation in prostate‐cancer resistant‐state transitions. The figure highlights the principal checkpoint‐related mechanisms, whereas additional context and evidence limitations are detailed in Section 8.3 and Table 5.

*Coordinates for this section*. Member: IFITM1 and IFITM3 (member‐resolved) · Compartment: immune‐cell‐intrinsic, myeloid (TAM, bone‐marrow), regulatory T cell and B cell, plus tumour‐cell immune surveillance · IFN‐input: mixed, type I IFN (STING–TBK1–IRF3), IFN‐γ–STAT1 (Treg) and IFN‐independent (B‐cell phosphatidylinositol 3,4,5‐trisphosphate (PIP3) scaffold) · Tumour‐milieu: LUAD, inflammatory bowel disease (IBD)‐associated CRC, syngeneic models, B‐cell leukaemia/lymphoma, brain metastasis · Tier ceiling: A (Treg‐IFITM3 and B‐cell‐IFITM3 anchors; see Table 5). Sections 8.1–8.3 are organised around three host compartments: the immune‐cell‐intrinsic compartment (this section), the stromal and microbiota‐conditioned compartment (Section 8.2), and the checkpoint/adaptive‐resistance compartment (Section 8.3). These three compartments are matched to the row groupings of Table 5 and to Figures 6 and 7. This section addresses the first of them. The preceding sections focused on tumour‐cell‐intrinsic IFITM biology, but cancer‐relevant IFITM function is not confined to transformed cells. Expression in myeloid effectors, lymphoid compartments and microenvironmental niches reshapes anti‐tumour immunity in ways tumour‐cell studies alone cannot capture, and the same family is repeatedly co‐opted by chronic inflammatory programs into adaptive immune resistance. The sign of effect is again member‐, compartment‐ and IFN‐quality‐specific. Table 5 provides a systematic summary of the evidence base for IFITMs across immune compartments, stroma and adaptive immune resistance (Figure 6).

Within tumour‐associated macrophages (TAMs), Tier B evidence shows that PLK1‐phosphorylated FoxM1 in LUAD activates STING–TBK1–IRF3 to upregulate IFITM1, which amplifies IL‐1/IL‐6/VEGFA expression, drives M2d‐like TAM polarisation, increases PD‐L1, and promotes metastasis.^128^ In melanoma, a CD133^+^PD‐L1^+^ subpopulation engages TGF‐β‐dependent niche stiffening to resist adoptively transferred engineered macrophages, a finding consistent with myeloid‐targeted therapies encountering checkpoint‐linked resistance.^129^ This study reports IFITM1 among the ISGs expressed by M1‐polarised macrophages in this setting, which places IFITM1 within the myeloid compartment of the resistance model; because IFITM1 is not itself perturbed, however, the evidence is marker‐level (Tier C) and does not establish that IFITM1 contributes causally to the observed resistance. Marker‐level Tier C evidence finds the murine‐specific paralog Ifitm6 induced in splenic, thioglycollate‐elicited and bone marrow‐derived macrophages of tumour‐bearing mice via lipopolysaccharide (LPS)/Toll‐like receptor (TLR) signalling, without functional demonstration and with no direct human counterpart.^130^ A computational signature ties AQP9 and IFITM1 to EMT, PI3K/AKT and immune infiltration in IBD‐associated CRC, requiring independent functional validation.^131^ As supporting non‐cancer evidence, IFITM1 drives macrophage‐like transdifferentiation of vascular smooth muscle cells in atherosclerosis via c‐Src/MAPK/GATA2/E2F2,^132^ reinforcing that IFITM1 can instruct macrophage‐like programs but explicitly not extending this finding to oncologic settings.

The strongest single piece of immune‐cell‐intrinsic IFITM evidence is Tier A: in tumour‐infiltrating FOXP3^+^ regulatory T cells, an IFN‐γ–STAT1–IFITM3 feedback loop maintains Treg fragility; IFITM3 depletion enhances STAT1 phosphorylation, drives a Th1‐like IFN‐γ‐secreting Treg phenotype and licenses anti‐tumour immunity.^65^ IFITM3 also functions as a PIP3 scaffold amplifying PI3K signalling at the B‐cell surface upon B‐cell receptor (BCR) engagement or oncogenic kinase activity, with prognostic relevance in B‐cell leukaemia and lymphoma.^58^ For NK biology, the IFN‐input axis itself matters: IRF3 controls poly‐I:C‐induced NK Granzyme B, IFN‐γ secretion and B16 melanoma control,^133^ evidence for the upstream IFN response, not for direct IFITM function. Tier C findings extend this picture: IFITM1 upregulation in bone marrow mononuclear cells correlates with severe cytokine release syndrome after CD19 chimeric antigen receptor T cell (CAR‐T) in B‐cell acute lymphoblastic leukaemia (B‐ALL), and IFITM1 knockdown reduces IL‐6 in co‐culture^134^; a bioinformatics signature links IFITM1 to tumour‐infiltrating immune cells in prostate adenocarcinoma.^135^

A compartment‐specific surveillance mechanism emerges in brain metastasis. Tier B in vivo CRISPR screening shows that IFITM1 on metastatic lung cancer cells secretes complement C3 to activate microglia and elevates MHC‐I to enhance CD8^+^ T‐cell killing, suppressing brain colonisation and correlating with checkpoint‐blockade response.^59^ These immune‐cell‐intrinsic functions are pro‐immunogenic in direction; the opposing, chronic‐inflammatory arm is developed in Section 8.3, after the stromal and microbiota inputs that condition it (Section 8.2).

### 8.2 Stroma, MSCs, microbiome and single‐cell TME views

*Coordinates for this section*. Member: IFITM1 and IFITM3 (member‐resolved; whole IFITM‐locus in the colitis model) · Compartment: stromal/host, cancer‐associated fibroblasts, mesenchymal stem cells, gut microbiota and epithelium, single‐cell TME · IFN‐input: conditioning by stromal and microbiota‐derived signals, type I IFN (CAF‐derived IFN‐β) and SCFA‐mediated activation of the JAK1–STAT1/2 pathway (microbiota‐derived signalling); otherwise stromal‐conditioned · Tumour‐milieu: breast (CAF), HCC (mesenchymal stem cells (MSCs)), ccRCC (microbiota), colitis‐associated cancer, gastric TME · Tier ceiling: B. Note directional divergence: IFITM3 is anti‐inflammatory/protective in the colitis context (see Table 5). Building on the immune‐cell‐intrinsic biology of Section 8.1, the second host compartment concerns non‐tumour cells that condition the IFN‐input axis itself. Stromal cells, MSCs and microbiota‐conditioned host cells do not act as IFITM effectors in the classical sense, but they set the magnitude, quality and duration of IFN signalling that downstream tumour cells receive, and therefore the level and behaviour of tumour‐cell IFITMs.

*Context‐only stromal evidence is limited but coherent*. A heterotypic in vitro model identifies a subset of IFN‐positive CAFs in breast cancer that drives proliferation of MCF‐7 cells in an IFN‐β‐dependent manner and tracks with poor prognosis in independent cohorts^136^; because this study does not measure or manipulate any IFITM, it is annotated as context only in Table 5 rather than assigned an IFITM evidence tier, and it defines a stromal source of type I IFN to which tumour‐cell IFITMs would respond rather than demonstrating an IFITM‐dependent effect. Tier C MSC evidence shows that adipose‐tissue‐derived MSCs enhance radiotherapy efficacy in HCC preclinical models,^137^ indicating that mesenchymal compartments can influence treatment response, although mechanistic IFITM linkage remains indirect.

Preliminary microbiome evidence (Tier B but restricted to two studies) points to microbiota‐conditioned shaping of the IFN/IFITM axis. Lachnospiraceae‐derived propionate inhibits ccRCC progression through short‐chain fatty acid (SCFA)‐mediated modulation of host inflammatory and IFN‐relevant programs,^138^ and anti‐inflammatory IFITM gene knockout exacerbates colitis while partially de‐protecting from colitis‐associated tumourigenesis via coordinated immunity and microbiota changes.^63^ These two reports are not directly contradictory: they address different organs and readouts, microbial propionate modulating IFN‐relevant programs in ccRCC^138^ versus whole‐locus IFITM knockout altering colitis and colitis‐associated tumourigenesis in the gut,^63^ rather than opposite directions of a single IFITM effect. Read through the framework, both describe the microbiome acting on the IFN‐input axis within the host/microbial‐interface compartment, with the sign of the downstream IFITM effect set by tissue and disease stage rather than fixed. Two limitations bound their interpretation: the colitis study manipulates the entire IFITM locus without member resolution and so cannot attribute its phenotype to IFITM1 specifically, and neither study measures tumour‐cell‐intrinsic IFITM output directly. These reports therefore cannot support broad causal claims and are best read as hypothesis‐generating evidence that the microbiome conditions the IFN/IFITM axis. At single‐cell resolution, dissection of oncofetal reprogramming in the gastric TME further shows that IFITM‐pathway features stratify stromal and immune states across patient samples.^26^

### 8.3 IFITMs, immune checkpoints and adaptive immune resistance

*Coordinates for this section*. Member: IFITM2 and IFITM3 (member‐resolved) · Compartment: transformed tumour cell (checkpoint/T‐cell engagement) · IFN‐input: chronic inflammatory (H3K27ac–TNF‐α/NF‐κB; JAK/STAT3) · Tumour‐milieu: TNBC, gastric, prostate (post‐androgen‐signalling inhibition), cancer‐stem‐cell contexts · Tier ceiling: A (TNBC IFITM3–PD‐L1 anchor; see Table 5). Building on Sections 8.1 and 8.2, this section addresses the checkpoint/adaptive‐resistance compartment: chronic inflammatory signalling co‐opts IFITMs into checkpoint engagement, generating adaptive immune resistance that mirrors the acute‐IFN‐driven, MHC‐I‐linked immunogenicity of Section 7. The effect is member‐, context‐ and IFN‐quality‐specific.

The strongest single anchor is Tier A. In TNBC, enriched H3K27ac at the IFITM3 promoter drives transcriptional upregulation of IFITM3, which activates the TNF‐α/NF‐κB axis to elevate PD‐L1, promote proliferation, migration, invasion, xenograft growth and resistance to anti‐PD‐1 therapy in vivo; TNF‐α/NF‐κB blockade reverses these effects, and IFITM3 knockdown restores anti‐PD‐1 sensitivity with increased CD8^+^ T‐cell infiltration.^61^

Tier B evidence extends the pattern. In gastric cancer, tumour‐derived IFITM2 induces PD‐L1 via JAK/STAT3, drives T‐cell apoptosis in co‐culture and xenografts, and correlates with lymphatic metastasis, advanced stage and poor survival.^122^ Tumour‐intrinsic SLC25A1 imposes a dual regulation, simultaneously activating a mitochondrial‐to‐nuclear cyclic GMP–AMP synthase (cGAS)–STAT1 type I IFN response and stabilising PD‐L1 through a fumarate–Keap1 axis, generating an inflamed TME that paradoxically retains vulnerability to PD‐L1 blockade (preprint; not peer‐reviewed)^139^; this study does not name or manipulate an IFITM and is cited only to frame the IFN‐input/PD‐L1 context, not as direct IFITM evidence. In prostate cancer, a study presented at the AACR Annual Meeting 2026 (conference abstract; Tier D) reported that neoadjuvant androgen‐ signalling inhibition promotes the emergence of mixed basal/club‐like cancer‐identity cells with upregulated MHC class I/II genes and ISGs, including IFITM1, IFITM2 and IFITM3. Because this study is not IFITM‐centred and the IFITM signal appears within a broader immunomodulatory transcriptional program, it is cited here only to contextualise IFITM/ISG features in therapy‐associated resistant‐state transitions.^140^ The chronic‐IFN arm of this axis operates within a broader regulatory architecture that is not IFITM‐specific but that constrains how these IFITM–PD‐L1 findings should be read. PD‐L1 output is set well upstream of any IFITM: in PD‐L1/PD‐L2‐high tumour cells the *CD274*/*PDCD1LG2* locus lies within a reorganised topologically associating domain (chr9: 5.4–5.6 Mb) containing a super‐enhancer that drives synchronous PD‐L1 and PD‐L2 transcription and permits recruitment of STAT3 and IRF1, with additional control exerted post‐transcriptionally through the long PD‐L1 3′UTR and post‐translationally through glycosylation, ubiquitination and exosomal export.^141^ This is architecturally parallel to the H3K27ac enrichment in TNBC^61^: sustained inflammatory signalling remodels chromatin at both the IFN‐stimulated effector locus and the checkpoint locus, so concurrent elevation of IFITM3 and PD‐L1 in the same tumour does not by itself establish that one is the proximal cause of the other. The same caution applies to the transcription‐factor arm. The IFITM2–JAK/STAT3–PD‐L1 axis described in gastric cancer^122^ is one instance of a general STAT3‐dependent route to PD‐L1‐mediated immune escape: pharmacological suppression of STAT3 signalling in TNBC cells lowers p‐STAT3 and PD‐L1, restores IFN‐γ and IL‐2 production by co‐cultured CD8^+^ T cells, reduces CD8^+^ T‐cell apoptosis, and is reversed by a STAT3 activator.^142^ Whether IFITM2 is functionally required for this route, or is co‐regulated alongside PD‐L1 downstream of the same STAT3 input, has not been tested. Because chronic‐IFN‐driven PD‐L1 is a pharmacologically accessible node, it can also be lowered by small‐molecule rather than antibody‐based means,^143^, ^144^ the intervention format most compatible with the compartment‐ and IFN‐quality‐selective strategy developed in Section 10.2. None of these studies examines IFITM proteins directly; they are cited to define the chronic‐IFN/STAT3/PD‐L1 boundary within which the IFITM findings above are interpreted, not as IFITM‐specific evidence. IFITM biology in cancer cannot be understood from tumour‐cell studies alone: immune‐cell‐intrinsic IFITM3 (most clearly in Tregs), stromal and microbiota‐shaped IFN‐input, and chronic‐inflammatory IFITM scaffolding of PD‐L1 (most clearly in TNBC) collectively explain why the same family can support immunogenicity in one context and adaptive resistance in another (Figure 7).

## 9 RECONCILING CONFLICTS: WHEN IFITMS SWITCH SIDES

The IFITM cancer literature is often described as contradictory: the same protein is reported as pro‐tumour in one setting and anti‐tumour in another, immunosensitising in one cancer and immunotherapy‐resistance‐promoting in another, host‐protective in colitis but tumour‐cell‐promoting in CRC. The argument advanced here is that these conflicts are not noise but diagnostic readouts of the four‐axis framework introduced earlier in this manuscript, member identity, IFN‐input quality, cellular compartment and tumour milieu, and that each apparent contradiction can be reformulated as a defined, testable hypothesis. None of the resolutions offered below is presented as established; each is a conjecture whose discriminating experiment is specified.

The first conflict concerns IFITM1 direction across epithelial cancers. The Section 3 literature supports a pro‐tumour role for IFITM1 across colorectal,^16^, ^17^ breast,^48^, ^68^ lung,^69^, ^70^ head‐and‐neck,^71^ laryngeal,^72^ and pancreatic^23^ carcinomas, where it scaffolds EMT, STAT, NF‐κB and CAV‐1‐dependent programs. Several reports run in the opposite direction: the Section 3.2 neurofibromatosis type 1‐associated malignant peripheral nerve sheath tumour (NF1‐MPNST) observation, in which IFITM1 is downregulated in malignant tumours and IFITM1 restoration restrains Ras–ERK activation^76^; a focused report in cervical squamous cell carcinoma showing that IFITM1 is downregulated and acts in a growth‐inhibitory manner consistent with its evolutionary antiviral role^75^; and the IFITM1‐dependent immune surveillance of brain‐metastatic cells, in which IFITM1 acts in a host‐protective direction.^59^ Within the framework, the member axis (IFITM1) and the compartment axis (tumour cell) are held constant; the variable is the tumour‐milieu axis. The reconciling hypothesis is that HPV oncoprotein context determines whether IFITM1's surface and signalling partners adopt a transformation‐restrictive or permissive configuration, that is, whether its antiviral program dominates or is overwritten by EMT/NF‐κB/STAT co‐option. The discriminating experiment is to transduce HPV E6 and E7 oncoproteins into a non‐cervical, IFITM1‐dependent epithelial line and to measure the IFITM1‐knockdown phenotype before and after; the hypothesis predicts that E6/E7 expression will flip the sign of the knockdown effect. Both opposing findings carry a control‐tissue caveat that tempers the conflict. IFITM1 is scored as ‘downregulated’ relative to benign plexiform neurofibromas in NF1‐MPNST^76^ and relative to normal or adjacent cervical epithelium in cervical SCC^75^; in each case the comparator (a benign nerve‐sheath lesion; virally exposed, IFN‐conditioned mucosa) may carry an unusually high baseline IFITM1, so the apparent tumour‐associated loss could partly reflect an elevated control baseline rather than a true reversal of IFITM1's direction. This does not negate the growth‐inhibitory data, but the magnitude of the ‘switch’ may be overstated and should be confirmed against IFITM1 levels in matched, lineage‐appropriate normal tissue.

The second conflict concerns IFITM3 direction in immunotherapy response. The Section 7 evidence places IFITM3 as a positive determinant of MHC‐I expression and anti‐PD‐1/PD‐L1 response in SCLC,^50^ reinforced by a pan‐cancer association between IFITM3 and an inflamed, immuno‐hot TME,^126^ whereas the Section 8.3 anchor places IFITM3 within an H3K27ac‐driven TNF‐α/NF‐κB/PD‐L1 axis that confers checkpoint resistance in TNBC.^61^ Member identity is constant. The variable is dual: the IFN‐quality axis differs (acute, IFN‐γ/NLRC5‐driven IFITM3 in SCLC versus chronic, NF‐κB/H3K27ac‐driven IFITM3 in TNBC), and the tumour‐milieu axis differs (neuroendocrine SCLC with preserved antigen‐presentation machinery versus chronically inflamed TNBC). This same chronic, NF‐κB‐coupled checkpoint logic is mirrored by IFITM2 acting through a JAK/STAT3/PD‐L1 axis in gastric cancer,^122^ indicating that the resistance arm is not idiosyncratic to IFITM3 or to TNBC. Two caveats constrain this attribution. First, IFN quality and tumour lineage co‐vary across these studies and cannot be separated from the existing data: neuroendocrine SCLC (with intact NLRC5‐driven antigen presentation) and epithelial, chronically inflamed TNBC differ in many respects beyond the acute‐versus‐chronic character of their IFN input, so the sign difference may reflect lineage‐ and model‐specific biology as much as IFN timing. The acute/chronic account is therefore one of several non‐separable hypotheses, not an established cause. Second, because of this confound the discriminating experiment must hold lineage constant: within a single isogenic model, IFITM3 direction should be tested under controlled acute‐versus‐chronic IFN exposure and defined NF‐κB input, rather than by cross‐comparing SCLC with TNBC. The prediction is that, with lineage fixed, chronic/NF‐κB‐coupled input shifts IFITM3 towards PD‐L1‐associated resistance while acute IFN‐γ input shifts it towards MHC‐I‐linked immunogenicity; paired single‐cell ATAC plus RNA‐seq (H3K27ac at the IFITM3 locus vs. transcript level) then serves as the readout. Independently of mechanism, IFITM3 transcript abundance alone is not interpretable as a checkpoint‐response biomarker.

The third conflict concerns IFITM compartment direction in colitis‐associated CRC. The Section 8.2 colitis study reports that anti‐inflammatory IFITM gene knockout exacerbates colitis and partially de‐protects from colitis‐associated tumourigenesis, implying a host‐protective IFITM role in early disease.^63^ The Section 3.1 CRC literature, by contrast, places IFITM1 as pro‐tumour in established CRC,^16^, ^145^ a direction independently recapitulated in IBD‐associated CRC, where IFITM1 is computationally tied to EMT, PI3K/AKT and immune infiltration.^131^ Here the member axis is held essentially constant, and the decisive variable is the compartment axis: IFITM activity in the host immune and microbial‐interface compartment of early colitis differs in sign from IFITM activity in the transformed epithelial compartment of established CRC. The reconciling hypothesis is that the same family member can be host‐protective and tumour‐cell‐promoting at the same anatomical site, with sign determined by which compartment expresses it. A second variable co‐varies with compartment here and should be acknowledged: inflammatory temporality. The host‐protective observation derives from a DSS/azoxymethane (AOM)‐DSS colitis model,^63^ an acute‐to‐subacute chemically induced injury, whereas the pro‐tumour epithelial signal derives from established and IBD‐associated (chronically inflamed) CRC.^16^, ^131^, ^145^ Acute and chronic intestinal inflammation differ in IFN quality (transient vs. sustained) as well as in which compartment predominantly expresses IFITM, so the colitis‐versus‐CRC sign flip may reflect the IFN‐input axis in addition to the compartment axis. The discriminating experiment should therefore span both regimes: compartment‐conditional (epithelial‐ vs. haematopoietic‐specific) IFITM1 knockout tested in an acute (DSS) and a chronic (recurrent‐DSS or IL‐10^−^/^−^) colitis‐associated CRC model, with the prediction that epithelial‐specific loss reduces tumour burden preferentially under chronic inflammation while haematopoietic‐specific loss increases early tumourigenesis preferentially under acute injury.

**FIGURE 8:**
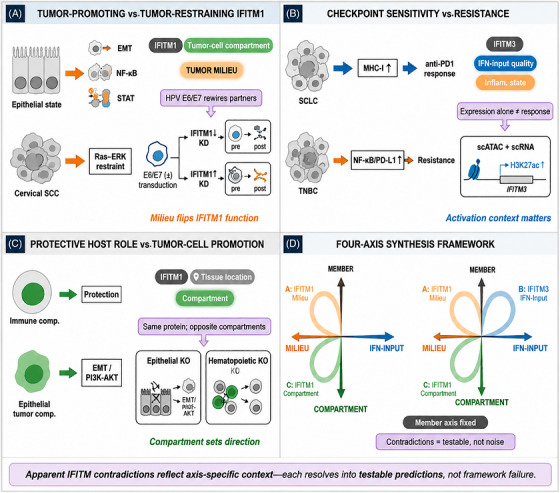
Apparent contradictions in interferon‐induced transmembrane (IFITM) function resolve along four contextual axes. Tumour milieu flips IFITM1 activity in epithelial cancers: pro‐tumour via EMT/NF‐κB/STAT in most carcinomas versus Ras–ERK restraint in cervical squamous cell carcinoma (SCC), for which HPV E6/E7‐mediated partner rewiring is presented as a testable hypothesis rather than an established mechanism (A). IFN‐input quality is the proposed discriminating axis for IFITM3 roles in checkpoint immunotherapy, acute, major histocompatibility complex class I (MHC‐I)‐driven sensitivity in small‐cell lung cancer (SCLC) versus chronic, NF‐κB/programmed death‐ligand 1 (PD‐L1)‐driven resistance in triple‐negative breast cancer (TNBC), but tumour lineage (SCLC vs. TNBC) co‐varies and cannot be separated from IFN‐input in the existing data (B). Cellular compartment is the proposed discriminating axis for IFITM1 in colitis‐associated CRC, host‐protective in immune cells versus EMT/PI3K‐AKT promotion in epithelial tumour cells, with inflammatory temporality (acute vs. chronic) co‐varying as a second axis (C); the four‐axis framework (D) turns these conflicts into testable predictions, not noise.

Across the three conflicts, the member axis is held constant while one or two of the remaining axes supply the discriminating variables; where two axes co‐vary and cannot be separated from existing data (as in the SCLC/TNBC and colitis/CRC conflicts), we say so explicitly and specify experiments designed to isolate them. This pattern is not a post hoc rationalisation: the conflicts cluster precisely where the framework predicts, and each is falsifiable by a single defined experiment. The strongest existing immune‐cell‐intrinsic finding, the IFN‐γ–STAT1–IFITM3 feedback loop in regulatory T cells,^65^ sits orthogonally to all three conflicts because it concerns a different compartment, illustrating that the framework operates simultaneously along multiple axes rather than reducing to any single one. Figure 8 maps the three documented conflicts onto the framework, showing that the member axis is held constant in each while one or two of the remaining three axes supply the discriminating variable, a single axis in the epithelial IFITM1 conflict, and two co‐varying axes in the SCLC/TNBC and colitis/CRC conflicts.

None of the reconciling hypotheses has been tested. The cervical SCC anti‐tumour finding rests on a small body of work^75^; the colitis observation is mouse‐model evidence^63^; and the TNBC versus SCLC contrast has not been examined within a single integrated study. The discriminating experiments described above are technically tractable but resource‐intensive, and any single experiment may resolve only one axis rather than the conflict in full. Cross‐comparison of findings remains constrained by heterogeneity in measurement (transcript vs. protein vs. chromatin state) and by the rarity of paired pre‐ and post‐treatment human samples for IFITM‐relevant analyses.

## 10 CLINICAL TRANSLATION, THERAPEUTIC STRATEGIES AND FUTURE DIRECTIONS

### 10.1 Biomarkers, imaging and predictive models

Across the four‐axis framework and the adaptive‐resistance synthesis of Section 8, translational maturity of IFITM biology is concentrated in biomarker discovery and preclinical imaging rather than therapeutic intervention. No IFITM‐targeted agent is in clinical trial, and the most defensible near‐term application is biomarker‐driven stratification for existing therapies. At present, the evidence supports IFITMs as prognostic or associative biomarkers, predominantly Tier C–D, not as validated companion stratifiers or therapeutic targets; no IFITM‐directed intervention has been tested in an immune‐competent, treatment‐response setting. This ceiling reflects what each assay can establish: IHC localises protein and can be member‐specific only with paralog‐validated antibodies; bulk RNA reports abundance but neither compartment nor member resolution; single‐cell RNA sequencing (scRNA‐seq) resolves cell type and compartment but is limited for protein abundance and post‐translational state; spatial proteomics adds in situ localisation and neighbourhood context; and only functional perturbation, performed in a member‐specific and compartment‐conditional manner in immune‐competent models, can establish causality or predictive value. Expression surveys therefore cannot, by themselves, promote an IFITM from biomarker to stratifier or target.

Tier D evidence supports IFITM‐family expression as an early molecular marker in β‐catenin‐driven colorectal adenomas and carcinomas.^145^ Faecal IFITM1/IFITM2 mRNA detection shows high specificity for CRC screening, with improved sensitivity in combination.^146^ Machine‐learning models incorporating IFITM1 reach area under the curve (AUC) >.9 for CRC diagnosis,^147^ and a CTSK/C3/IFITM1 signature reaches AUC >.85 for *Helicobacter pylori*‐associated gastric cancer.^148^ IFITM1 also appears within a dermatomyositis‐signature for hepatocellular carcinoma risk prediction^149^ and a multi‐gene IFITM1/IMPA2/KCNN3 nomogram for sunitinib resistance and prognosis in ccRCC.^150^ Tier C imaging evidence is restricted to a single IFITM1‐targeted second near‐infrared window (NIR‐II) fluorescence probe (IRDye800CW) that visualises CRC tumours and metastatic lymph nodes in preclinical orthotopic models and ex vivo human specimens, with no in‐human imaging study yet reported.^151^ Almost all of these reports are single‐cohort studies and lack external validation.

Six structural limitations constrain interpretation, and each has a defined remedy. First, cell‐line and correlative omics studies dominate over immune‐competent in vivo work; this requires external validation cohorts and compartment‐conditional mouse models. Second, IFITM2, IFITM5 and IFITM10 remain under‐represented relative to IFITM1 and IFITM3; this requires member‐specific functional studies rather than family‐level surveys. Third, older studies frequently conflate family members; this requires paralog‐validated antibodies and member‐specific genetic reagents. Fourth, single‐cell and spatial resolution of tumour‐cell versus immune‐cell versus stromal IFITM expression remains limited; this requires paired single‐cell ATAC and RNA sequencing. Fifth, human‐cohort immunotherapy‐response data outside SCLC and TNBC are sparse; this requires prospective, biopsy‐anchored response cohorts. Sixth, IFN‐input duration and quality cannot currently be measured directly in human tumours; this requires validated surrogates, such as paired chromatin‐accessibility and transcript readouts at the IFITM3 locus.

Translational maturity sits at Tier C/D biomarker and imaging discovery with zero therapeutic readiness. Near‐term IFITM translation will be biomarker‐driven, not therapeutic; the literature does not support clinical‐trial‐readiness for any IFITM‐directed agent, and overstatement on this point would discredit the field.

### 10.2 RNA‐based and pathway‐directed targeting: Opportunities and constraints

The cancer‐relevant outputs of IFITMs are surface–signalling scaffolding (IFITM1) and endolysosomal kinase rewiring (IFITM3) rather than discrete enzymatic activities. This biology favours expression‐level interventions, short interfering RNA (siRNA), antisense oligonucleotides (ASOs), mRNA payloads delivered using lipid nanoparticles (LNPs), and miRNA mimics or inhibitors, over small‐molecule active‐site targeting, and favours pathway‐node intervention at JAK/STAT, NF‐κB, MET/AKT or bromodomain and extra‐terminal domain (BET) proteins/histone deacetylase (HDAC; for H3K27ac‐driven IFITM3) over direct IFITM engagement. Because the relevant pharmacology must intercept a scaffold rather than block an active site, the design space shifts from selective inhibitor chemistry to selectivity in delivery, dose and timing.

Existing preclinical anchors are sparse but instructive. The AML IONP–cytarabine study^102^ provides delivery and target‐modulation proof‐of‐principle by downregulating IFITM3 alongside cytarabine. The HCC microRNA and long non‐coding RNA work^92^, ^93^, ^94^ characterises natural regulators of IFITM3, the miR‐29a, TUG1 and CKMT2‐AS1/miR‐142‐5p axes, that could in principle be mimicked or inhibited, although none has progressed to therapeutic formulation. In AI‐resistant breast cancer, pathway‐directed re‐sensitisation through JAK/STAT inhibition or broader ISG targeting illustrates how IFITM1‐dependent endocrine resistance can be addressed at the pathway level rather than at the IFITM protein itself.^49^, ^79^, ^80^

Four constraints are not optional. First, IFITMs are broadly expressed in healthy tissues with physiological antiviral roles, so unrestricted knockdown risks immunosuppression and heightened viral susceptibility. Second, the four‐axis framework requires preserving acute‐IFN immunogenicity (Section 7) while disrupting chronic‐IFN/NF‐κB‐driven resistance (Section 8.3),^61^ demanding context‐selective rather than total targeting. Third, selective delivery to a defined compartment, tumour cell versus immune cell versus stroma, is not currently achievable at therapeutic specificity with available RNA‐payload technologies, and the Section 8 evidence makes clear that compartment misallocation could turn an intended anti‐tumour effect into immune suppression. Fourth, broader ISG off‐target effects are likely with any RNA‐based intervention and have not been systematically assessed.

A credible near‐term IFITM‐pathway clinical strategy is therefore not an IFITM‐directed monotherapy but a biomarker‐stratified combination: an established pathway inhibitor (JAK/STAT, MET or BET) added on top of checkpoint blockade in tumours selected by IFITM expression and by an IFN‐quality surrogate that distinguishes acute from chronic input, for example paired chromatin‐accessibility and transcript readouts at the IFITM3 locus that can be assessed in archival tumour biopsies. This is consistent with the current direction of checkpoint pharmacology. Small molecules directed against immune‐checkpoint proteins, PD‐L1 foremost among them, have advanced substantially since 2020 and offer dose, schedule and tissue‐penetration control that antibodies do not, although the field still depends largely on in vitro binding and reporter assays and only rarely on humanised in vivo models, with newer modalities such as PROTACs and peptides at an early stage.^144^ Repurposed agents illustrate the same node‐directed principle: the tetracyclic antidepressant maprotiline lowers PD‐L1 in B16 melanoma cells and increases splenic CD4^+^, CD8^+^ and NK‐cell proportions together with intratumoural CD4^+^ and CD8^+^ T‐cell infiltration in immune‐competent C57BL/6 mice.^143^ For an IFITM‐informed strategy the relevant point is that such agents act on the chronic‐IFN/PD‐L1 node rather than on IFITM proteins, for which no ligandable site has been defined; the corresponding limitation is that they are not compartment‐selective and none has been evaluated in an IFITM‐stratified setting. As above, these are checkpoint‐pharmacology references rather than IFITM‐specific evidence. This proposal is currently a hypothesis, not a developed program, and would require prospective IFN‐quality biomarkers, demonstration of selectivity for chronic over acute IFN signalling, and careful management of broader ISG off‐target effects across normal tissues.

### 10.3 Limitations and future directions

Compared with prior focused IFITM reviews, most notably one centred on pancreatic cancer,^152^ the synthesis advanced here extends the scope along three explicit dimensions: it introduces the four‐axis framework (member, IFN‐quality, compartment, tumour‐milieu) of Section 2, it reframes the IFITM literature around the corrected acute‐versus‐chronic IFN distinction of Section 6, and it converts the field's apparent contradictions (Section 9) into testable hypotheses rather than presenting them as residual noise. The pancreatic review consolidated tumour‐type‐specific evidence within one disease; this manuscript organises cross‐tumour evidence around shared mechanistic axes.

The limitations enumerated in Section 10.1 cluster into four strategic themes. First, evidence is dominated by cell‐line and correlative omics work; immune‐competent in vivo demonstrations are rare and currently restricted to the IFN‐γ–STAT1–IFITM3 Treg axis,^65^ the IFITM1‐mediated brain‐metastasis surveillance program,^59^ the H3K27ac–IFITM3–TNF‐α/NF‐κB/PD‐L1 axis in TNBC,^61^ and the MET/AKT/FOXO3/c‐MYC gastric IFITM3 anchor.^28^ Most prognostic claims rest on single cohorts without external validation.^145^, ^148^, ^150^ Second, the family is unevenly studied: IFITM1 and IFITM3 dominate, whereas IFITM2 (Section 5.1), IFITM5 and IFITM10 (Section 5.2) are underpowered, and older work conflates non‐equivalent members despite direct biochemical evidence of IFITM1/IFITM3 non‐equivalence (Section 2.1). Third, the IFN‐input quality axis cannot be measured directly in human tumours; chromatin‐state surrogates such as H3K27ac at the IFITM3 locus^61^ and paired chromatin–transcript readouts will be required to operationalise it. Fourth, and following from Section 6.1, it is not established whether individual IFITM members are functional constituents of the IRDS or merely co‐regulated readouts of the same chronic, low‐dose type I IFN input. Distinguishing these possibilities requires member‐specific perturbation within a defined IRDS‐high model, with DNA‐damage and checkpoint‐resistance phenotypes read out in parallel, and we identify this as the single highest‐priority mechanistic gap arising from the framework.

A defensible future‐directions program follows. Compartment‐conditional knockout models should be applied to the three Section 9 conflicts. Member‐specific reagents should replace family‐level treatments. Single‐cell and spatial profiling should extend to immunotherapy cohorts beyond SCLC (Section 7) and TNBC.^61^ Prospective cohorts should test whether IFITM expression combined with an IFN‐quality surrogate predicts checkpoint‐blockade response better than either alone, and paired pre‐ and post‐treatment biopsies should anchor IFITM‐relevant trials. The IFITM cancer field is rich in correlations^92^, ^93^, ^94^, ^145^, ^148^, ^150^ and increasingly precise in mechanism,^28^, ^59^, ^61^, ^65^ but its translational maturity remains constrained. Honest acknowledgment of these constraints, rather than overstatement of recent claims, is the prerequisite for any IFITM‐informed clinical strategy.

## 11 CONCLUSION

The IFITM family is best understood not as a single biological entity but as a context‐dependent set of cancer regulators whose meaning is set by member identity, the quality of upstream IFN signalling, the cellular compartment in which expression occurs, and the surrounding tumour milieu. This reading dissolves what is described as contradiction into testable predictions: that HPV oncoprotein context determines whether IFITM1 acts oncogenically or restrictively in epithelial cancers; that chromatin state at the IFITM3 locus, not transcript level, distinguishes responsive from resistant tumours; and that compartment‐specific IFITM expression can simultaneously protect the host and promote the transformed epithelium at one anatomical site. Each prediction can be falsified by a defined experiment, and none is presented here as established.

The evidence base sustaining this framework is uneven. The evidence base sustaining this framework is uneven. The strongest mechanistic anchors, the IFN‐γ–STAT1–IFITM3 feedback loop in regulatory T cells, the H3K27ac–IFITM3–TNF‐α/NF‐κB/PD‐L1 axis in TNBC, IFITM3‐driven MHC‐I induction in SCLC and IFITM3–MET/AKT scaffolding in gastric cancer, coexist with a larger body of single‐cohort, correlative and cell‐line evidence whose generalisability is unestablished. The IFITM2, IFITM5 and IFITM10 literatures remain underpowered relative to IFITM1 and IFITM3, and the IFN‐quality axis cannot yet be measured directly in human tumours; chromatin and transcript surrogates at the IFITM3 locus offer a tractable but unvalidated proxy.

Near‐term clinical translation will not be IFITM‐directed. The defensible application is biomarker‐stratified selection of patients for existing pathway inhibitors and checkpoint blockade, paired with IFN‐quality surrogates and external validation. Honest acknowledgment of these constraints, rather than overstatement of readiness, is the precondition for any IFITM‐informed clinical strategy and for the prospective experiments through which the four‐axis framework will either be substantiated or falsified. The field's most useful next step is not another expression survey but the conditional, compartment‐resolved, IFN‐quality‐stratified studies the framework demands.

## AUTHOR CONTRIBUTIONS

**Zhe Liu**: Conceptualisation; methodology; investigation; writing—original draft; writing—review and editing. **Jie Meng**: Methodology; investigation; writing—original draft. **Zengyan Lai**: Methodology; investigation; writing—original draft. **Fuqiu Li**: Methodology; investigation; writing—original draft. **Sha Lv**: Conceptualisation; supervision; methodology; writing—original draft; writing—review and editing. All the authors reviewed and approved the final manuscript.

## FUNDING INFORMATION

The authors received no specific funding for this work.

## CONFLICT OF INTEREST STATEMENT

The authors declare no conflicts of interest.

## ETHICS STATEMENT

Not applicable. This review did not involve new studies on human participants or animals; all data discussed derive from previously published, ethically approved studies.

## GENERATIVE AI STATEMENT

The authors declare that no generative artificial intelligence (AI) tools or AI‐assisted technologies were used during the preparation of this manuscript. The manuscript text, scientific interpretation, literature evaluation, evidence assessment and conclusions were entirely developed by the authors. All figures, graphical illustrations and visual materials were designed, prepared and edited by the authors using Adobe Illustrator without the use of AI‐based image‐generation tools. The authors take full responsibility for the accuracy, originality and integrity of the content presented in this manuscript.

## Data Availability

This review analysed no new data. All findings discussed derive from previously published studies, which are cited at the point of use.
